# A kinematically Bifurcated Metamaterial for Integrated Logic Operation and Computing

**DOI:** 10.1002/advs.202509829

**Published:** 2025-07-17

**Authors:** Kaili Xi, Jingsong Wei, Xiao Zhang, Jiayao Ma, Zhong You, Changqing Chen, Yan Chen

**Affiliations:** ^1^ Key Laboratory of Mechanism Theory and Equipment Design of Ministry of Education Tianjin University 135 Yaguan Road Tianjin 300350 China; ^2^ Department of Engineering Science University of Oxford Parks Road Oxford OX1 3PJ UK; ^3^ Department of Engineering Mechanics, CNMM and AML Tsinghua University Beijing 100084 China; ^4^ School of Mechanical Engineering Tianjin University 135 Yaguan Road Tianjin 300350 China

**Keywords:** kinematics, mechanical computing, mechanical metamaterials, system integration

## Abstract

Mechanical computing metamaterials, which utilize transitions among discrete configurations to process information autonomously when perceiving external stimuli, are important in the development of intelligent mechanical systems. However, most current designs involve tessellations of planar mechanisms or bistable structures, which typically offer only two distinct configurations, requiring many units to provide multiple input‐outputs for complex logic operations with little consideration of system integration. Here, We propose a family of 2*n*‐side kinematic polygonal modules introducing *n* decoupled inputs and 2*^n^* transitable extreme configurations, which are coupled with electrical circuits to construct conductive logic metamaterials for mechanical computing. To simplify the construction of logic computing systems, a minimized combinatorial logic canonical function, the parallel computing sum of the product (PCSoP) function, is developed. We first design and integrate seven basic logic gates on a quadrilateral module, followed by implementing all four types of 2‐bit arithmetic operations with a single polygonal module, where the 2‐bit divider on an octagonal module is designed for the first time. Moreover, information display and simultaneous recognition of three mathematical properties of the decoded decimal numbers 2‐15 are implemented on one polygonal module. This kinematics‐based design strategy for mechanical computing metamaterials will greatly advance the development of mechanical intelligence.

## Graphical abstract

A family of 2*n*‐side kinematic polygonal modules with *n* decoupled inputs and 2*^n^* extreme configurations via kinematic bifurcation is proposed. It allows integrating seven basic logic gates on a quadrilateral module. Moreover, a minimized Parallel Computing Sum of Products function is developed, enabling all 2‐bit arithmetic (including division), information display, and recognition on single modules of up to eight units.

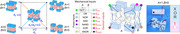

## 1 Introduction

Mechanical computing, overshadowed by electronic computing for about 100 years, has resurged in recent years because it provides a sensing‐computing‐actuating integrative deformation controlling strategy that facilitates the development of mechanical matter with embedded intelligence.^[^ ^1^, ^2^, ^3^, ^4^ ^]^ Correspondingly, the metamaterials utilizing the transitions among discrete configurations based on multi‐stable structures or mechanical mechanisms are developed to process information autonomously under external stimuli for various applications, including mechanical logic,^[^ ^5^, ^6^, ^7^ ^]^ computing,^[^ ^8^, ^9^, ^10^, ^11^ ^]^ and associated logical functionalities such as control,^[^ ^12^, ^13^, ^14^ ^]^ decision‐making,^[^ ^15^, ^16^ ^]^ and other functions.^[^ ^17^, ^18^, ^19^, ^20^ ^]^

To implement complicated information processing tasks, logic gates and their combinations of these gates must be designed^[^ ^1^, ^21^, ^22^, ^23^, ^24^ ^]^ For logic gates, digital inputs and outputs are first abstracted from the distinct configurations such as binary bits ‘0’ and ‘1’ are related to the two stable states of bistable origami,^[^ ^25^, ^26^, ^27^ ^]^ curved‐beam structures^[^ ^28^, ^29^ ^]^ or soft valves^[^ ^30^, ^31^ ^]^ units, or two extreme configurations of mechanism.^[^ ^32^, ^33^ ^]^ However, in current designs, each unit can be reconfigured into only two or three basic configurations.^[^ ^30^, ^34^, ^35^ ^]^ The resulting logic gates typically require at least two‐unit arrays to perform most logic operations. Regarding combinations of these gates, both parallel and series combinations of mechanical logic gates have been explored to perform different computing tasks.^[^ ^36^, ^37^ ^]^ Although it is theoretically feasible to directly combine many functional mechanical logic gates into large‐scale mechanical computing systems, limited by damping, dissipation, and potential energy barriers, signal propagation is constrained in the systems,^[^ ^1^, ^8^, ^27^, ^38^ ^]^ making it difficult to ensure that assemblies with a large number of mechanical units generate the predefined motion and yield the corresponding outputs.

To obtain simplified mechanical computing systems, a bottom–up design framework based on fundamental logic gates (NOT and buffer gates) has been proposed, which employs the Quine‐McCluskey (QM) method to merge neighboring minimal terms to simplify these Boolean functions into more compact QM sum of product (QMSoP) forms.^[^ ^23^ ^]^ However, due to lack of accessible interactions between gates, this framework has not yet been implemented in a minimalist structure without redundant units. Moreover, a top‐down design approach, which emphasizes the importance of the structural design and layout, has been employed to reduce system complexity by integrating a 2‐bit adder into a system with 10 mechanism units^[^ ^39^ ^]^ without assembling individual logic gates as the bottom–up design framework. However, the number of necessary units increases dramatically in scenarios with other 2‐bit arithmetic operations or information processing.^[^ ^39^ ^]^ Therefore, a comprehensive design strategy for both logic gate design and their combination is still needed for highly integrated mechanical computing metamaterials.

Here, inspired by the kinematic bifurcation of linkage assemblies, we propose a family of 2*n*‐side polygonal modules with 2*^n^* distinct configurations suitable for mechanical bit abstractions that are capable of accepting *n* decoupled inputs. We also develop a PCSoP function by introducing the concept of temporal parallelism from parallel computing (PC),^[^ ^40^, ^41^, ^42^ ^]^ which simplifies the QMSoP function through extracting duplicate mechanical logic network components as a single sub‐task. By exploiting the wide variety of module configurations and PCSoP functions, we have achieved an unprecedented level of system integration in mechanical computing, including integrating all seven basic logic gates in a single quadrilateral module; realizing arithmetic operators including adder, subtractor, and multiplier with a substantially reduced number of switches; and implementing both the 2‐bit divider and simultaneous recognition of three mathematical properties of the decoded decimal numbers 2‐15 on an 8‐unit octagonal module for the first time. This work shows great potential of integrated mechanical metamaterials for advanced arithmetic operations, intelligent responses, encryption, and communication.

## 2 Results

### 2.1 Kinematically Bifurcated Polygonal Modules and Mechanical Bit Abstractions

**Figure 1:**
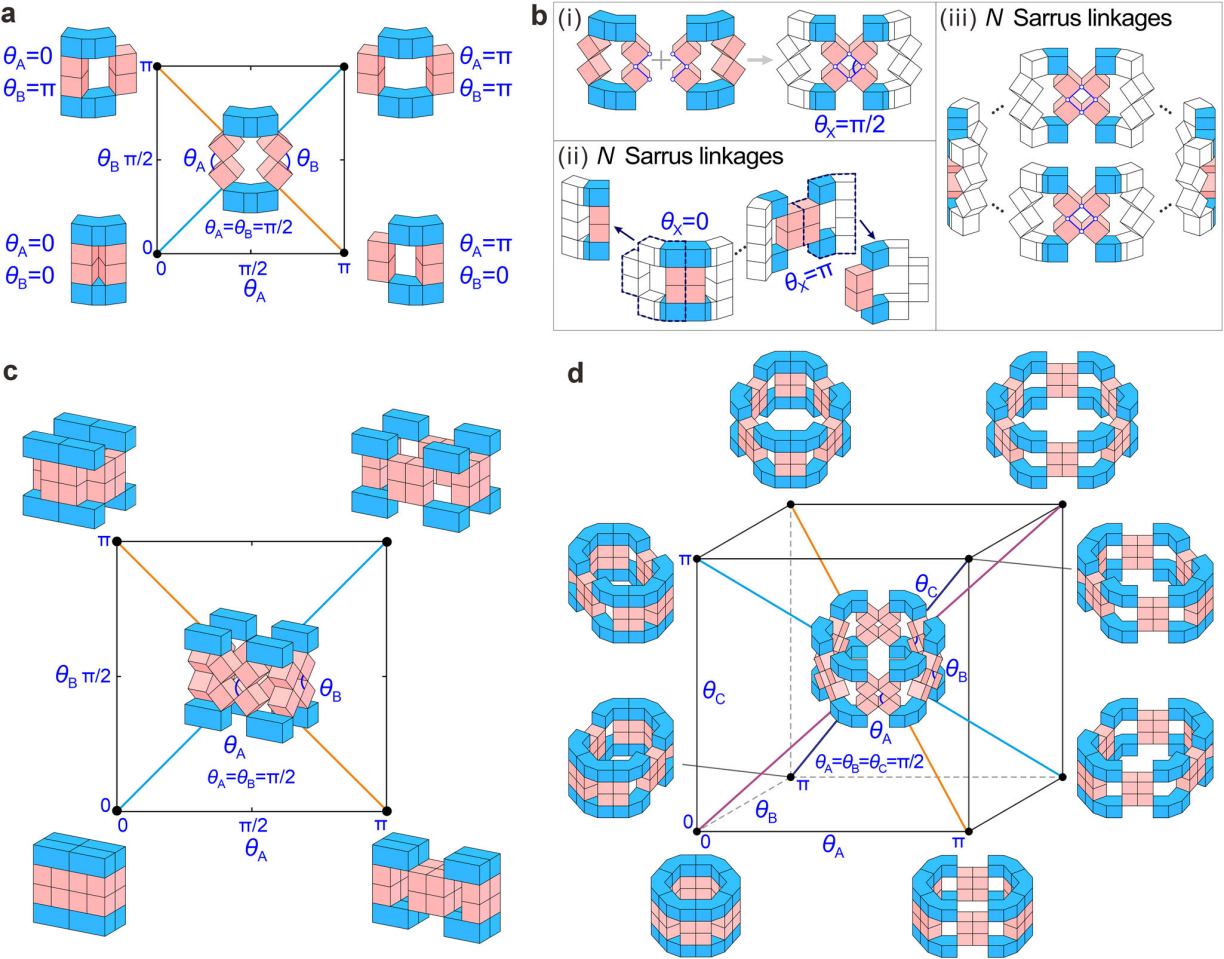
Construction and kinematic bifurcation of polygonal modules. a) Sarrus linkage with a kinematic bifurcation point at *θ*_A_ = *θ*_B_ = π/2, allowing transitions among four extreme configurations at *θ*_A_ = *θ*_B_ = 0, *θ*_A_ = 0 & *θ*_B_ = π, *θ*_A_ = π & *θ*_B_ = 0, and *θ*_A_ = *θ*_B_ = π, where *θ*_A_ and *θ*_B_ are dihedral angles between the left and right pairs of pink cubes. The blue blocks (formed by two cubes and a triangular prism) are connected by pink cubes, and the blue and orange lines indicate two motion paths of the Sarrus linkage. b(i) An assembly of two Sarrus linkages connected by a 4‐bar linkage. b(ii) Construction of an open‐loop metamaterial with *N* Sarrus linkages connected by *N*‐1 4‐bar linkages, where two extreme configurations of the 4‐bar linkage at *θ*_X_ = 0 and π. b(iii) Construction of a closed‐loop polygonal module with *N* Sarrus linkages connected by *N* 4‐bar linkages. c) Kinematic bifurcation behavior of a quadrilateral module, transitioning through the kinematic bifurcation point with four extreme configurations (*θ*_A_ = *θ*_B_ = 0, *θ*_A_ = 0 & *θ*_B_ = π, *θ*_A_ = π & *θ*_B_ = 0, and *θ*_A_ = *θ*_B_ = π). d) Hexagonal module with three decoupled angles *θ*_A_, *θ*_B_, and *θ*_C_ and eight extreme configurations.

For mechanical bit abstractions, we propose a family of polygonal module metamaterials that can switch between multiple motion paths due to kinematic bifurcation, thereby achieving many distinct configurations. We first consider a Sarrus linkage^[^ ^43^ ^]^ with one DOF which has a bifurcation configuration at *θ*_A_ = *θ*_B_ = π/2 with two possible motion paths, *θ*_B_ = *θ*_A_ (path I, the blue line) or *θ*_B_ = π − *θ*_A_ (path II, the orange line), where *θ*_A_ and *θ*_B_ are dihedral angles between the two adjacent pink cubes (**Figure** **1**a). For convenience, we refer to the configurations where the pink cubes fit tightly together as extreme configurations, in which the two kinematic variables *θ*_A_ and *θ*_B_ can have values of 0 or π, resulting in 2^2^ sets of combinations for the configuration coordinate (*θ*_A_, *θ*_B_): (0, 0), (0, π), (π, 0), and (π, π). In this work, we consider such *θ*_X_ as the decoupled inputs, maintaining a one‐to‐one correspondence with extreme configurations. Two Sarrus linkages can be assembled through a 4‐bar linkage formed by two pink‐cube pairs from adjacent Sarrus linkages (Figure 1b i), and the 4‐bar linkage is characterized by an input *θ*_X_; such an assembly has only one DOF, with similar bifurcation behavior for each Sarrus linkage. *N* Sarrus linkages can be connected by *N*‐1 4‐bar linkages to form an open‐loop metamaterial following an irregular curve (Figure 1b ii). When all inputs *θ*_X_ to the 4‐bar linkages are set to 0 or π in the assembly, the metamaterial reaches an extreme configuration, but the Sarrus linkages at both ends still have two possible configurations due to bifurcation, making them unsuitable for mechanical bit abstractions. In addition, we also have explored the construction of straight‐line metamaterials using a similar assembly method. However, these straight‐line metamaterials either exhibit more DOFs, asynchronous motions between units, and unclear motion paths, rendering them unsuitable for mechanical bit abstractions, or only partial units are available for abstracting mechanical bits (see Section S1, Supporting Information, for details). When *N* Sarrus linkages are connected by *N* 4‐bar linkages (*N* > 2), an *N*‐sided closed‐loop polygonal module metamaterial can be formed (Figure 1b iii). A thorough kinematic analysis (Section S1, Supporting Information) reveals that any *N*‐sided polygonal module has one DOF. Since the closed‐loop module metamaterials can maintain a single DOF and allow transitions to various extreme configurations through kinematic bifurcation, they are selected for further analysis.

Subsequently, the effect of *N* on the bifurcation behaviors of the closed‐loop polygonal module metamaterials is explored. When *N* is a prime number, there is no bifurcation in the whole motion path, and the polygonal module has only one input and two extreme configurations. However, when *N* is a composite number, i.e., *N* = *m* × *n*, the polygonal module can be divided into *m* identical groups on the basis of each factor *n* (excluding *n* = 1 and *N*). In this case, each *θ* in the group corresponding to factor *n* is decoupled (Section S1, Supporting Information), leading to *n* decoupled inputs. Consequently, 2*^n^* ^−1^ kinematic paths exist in the bifurcation configuration with all *θ_i_* = π/2 (*i* = 1, 2, …, *N*), resulting in 2*^n^* extreme configurations. Especially, for polygonal modules with an even number of sides, *m* = 2 is preferred to achieve the largest possible *n*; that is, we can obtain the maximum number of decoupled inputs (*n*) from the same polygonal modules and the maximum number (2*^n^*) of extreme configurations. This fascinating kinematic bifurcation behavior makes single‐DOF 2*n‐*polygonal modules ideal base materials for conductive digital logic structures.

In particular, the mechanical bit is defined as follows: when *θ*_X_ = 0, the mechanical bit input *X* = 0 corresponds to the configuration where the top and bottom blue blocks are closed; when *θ*_X_ = π, *X* = 1, and the blue blocks are separated. For two mechanical bit inputs (*A*, *B*) to the quadrilateral module, four combinations corresponding to four distinct configurations can be realized: (0, 0), (0, 1), (1, 0), and (1, 1) (Figure 1c). When 2*n* = 6, the hexagonal module has eight (2^3^) configurations with three decoupled inputs (Figure 1d). Similarly, when 2*n* = 8, the octagonal module has 2^4^ configurations with four decoupled inputs (Figure S2, Supporting Information). Hence, a kinematically bifurcated, single‐DOF, 2*n*‐polygonal module can accept *n* decoupled signal inputs and achieve 2*^n^* extreme configurations, thereby establishing a scalable substrate for binary‐state mechanical computing.

### 2.2 The Quadrilateral Logic Module Integrating Seven Basic Logic Gates

**Figure 2:**
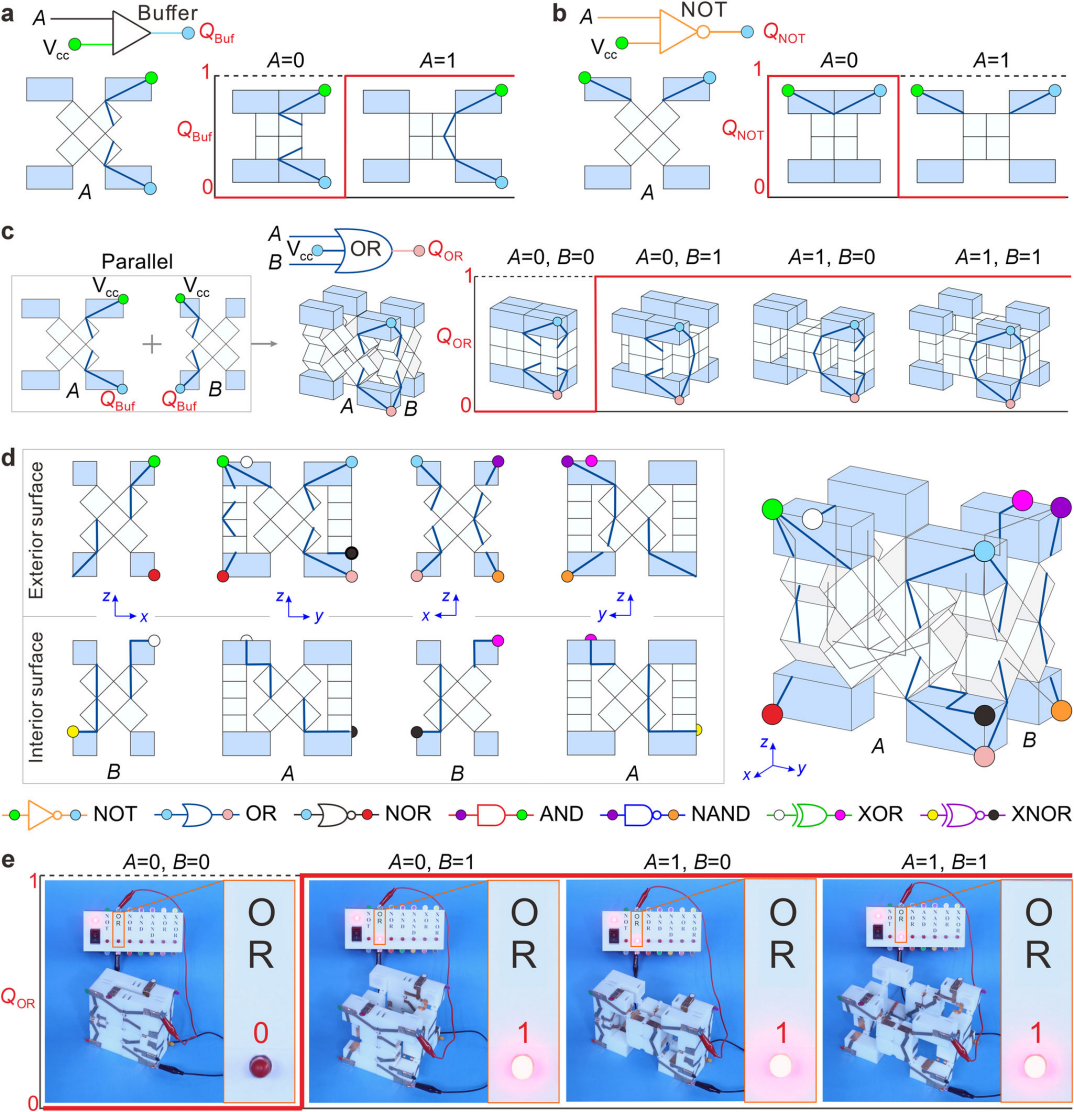
Design, integration, and experiments with the logic quadrilateral module. a) Schematic of the buffer gate on the front surface of the quadrilateral module, with electrical output *Q*_Buf_ = 0 for mechanical input *A* = 0 and *Q*_Buf_ = 1 for *A* = 1. The power input terminal is V_cc_. b) Schematic of the NOT gate, representing an inverse electrical switch. c) Schematic of the OR gate formed by two buffer gates in parallel with mechanical inputs *A* and *B* and electrical output *Q*_OR_. d) A single quadrilateral module integrating all seven basic logic gates: NOT, OR, AND, NOR, and NAND gates on the exterior surfaces of the module and XOR and XNOR gates on the interior surfaces. The input and output nodes of these logic gates are denoted by different colored dots, as illustrated in the schematic of each logic gate at the bottom. e) Experimental outputs (red text) of the OR logic gate on the integrated module with different mechanical inputs (black text).

Coupling these polygonal module metamaterials with electrical circuits, we construct a set of conductive logic modules for various logic operations. Taking the quadrilateral module as the basic module for designing the buffer gate, the power input terminal and electrical output nodes are V_cc_ and *Q*_Buf_, represented by the green and light blue dots, respectively, in **Figure** **2**a. Circuits (marked as navy lines) are attached to the front surface of the quadrilateral module (where angle *θ*_A_ is located, Figure 1c) to implement a switch consistent with the buffer logic. For input *A* = 0 with module configuration *θ*_A_ = 0 (i.e., the circuit is not connected), the output *Q*_Buf_ is 0. When *A* = 1 with *θ*_A_ = π (i.e., the circuit is connected), *Q*_Buf_ = 1. The NOT logic gate (Figure 2b) is the inverse of the buffer gate, with the logical formulation *Q*_NOT_ = $\bar{A}$. For input *A* = 0 with module configuration *θ*_A_ = 0 (i.e., a connected circuit), the output *Q*_NOT_ is 1. Conversely, when *A* = 1 and *θ*_A_ = 0 (i.e., an unconnected circuit), *Q*_NOT_ = 0. These are just two examples of circuit designs among many possible solutions; see Section S2, Supporting Information, for details on other feasible circuit arrangements and for designs driven by input *B* (on the surface of the quadrilateral module where angle *θ*_B_ is located, Figure 1c).

As the above two fundamental logic gates require a single mechanical input, their circuits can be located on one surface of the quadrilateral module. However, two inputs *A* and *B* must be considered for the six dual‐input logic gates, i.e., OR, NOR, AND, NAND, XOR, and XNOR, and the corresponding circuits cover two or more module surfaces. For example, the OR gate shown in Figure 2c implements the logical operation *Q*_OR_ = *A*+*B* with mechanical inputs (*A*, *B*); this gate is formed by connecting two buffer gates in parallel on two adjacent surfaces of the quadrilateral module (here, the front and right surfaces are used as an example; however, the gates can be placed on any two adjacent surfaces). The OR gate outputs a value of 1 when at least one of the inputs is 1, i.e., there is at least one connected line. All the other logic gates can be constructed using parallel and/or series connections of buffer and/or NOT gates (Figure S5a–e, Supporting Information).

As each facet of this module not only has two inner and outer facets, but also four inner facets and four outer facets forming a closed loop, respectively, this provides more possibilities for the layout of circuits to realize integrating multiple logic gates on a module, thereby increasing its computational utility. We next explore the feasibility of integrating a single‐input gate (NOT) and six dual‐input logic gates (OR, NOR, AND, NAND, XOR, and XNOR) into one quadrilateral module. If each switch occupies a unique module surface, all 17 switches for the seven logic gates cannot be integrated into the eight surfaces (four exterior and four interior surfaces) of the quadrilateral module. Hence, we must ensure that some switches share the same surfaces or that some logic gates share the same switches. As illustrated in Figure S5g (Section S3, Supporting Information), the NOT, OR, NOR, AND, and NAND logic gates are integrated on the four exterior surfaces of the quadrilateral module utilizing shared surfaces and partial circuits. Additionally, by sharing switches, the XOR and XNOR gates are implemented within the module using four switches across the four interior surfaces (Figure S5g, h in Section S3, Supporting Information). Obviously, these logic gates located on the exterior and interior surfaces can change positions with each other. By combining overlapping circuits into a single circuit (Figure 2d), we integrate all seven gates into a single quadrilateral module, see Section S3, Supporting Information for the design details. The prototype of the integrated module was fabricated and experimentally assessed. For example, the OR gate on the integrated module provides an experimental output *Q*_OR_ = 0 when both inputs *A* and *B* are 0, and *Q*_OR_ = 1 for all other combinations (Figure 2e). LED states indicate the outputs (1 = on, 0 = off), and experimental results for the other logic gates are shown in Figure S6 (Supporting Information). By selectively engaging input and output nodes corresponding to different logic gates, the integrated module executes all seven basic logic gates (Movie S1, Supporting Information).

### 2.3 Arithmetic Operations

**Figure 3:**
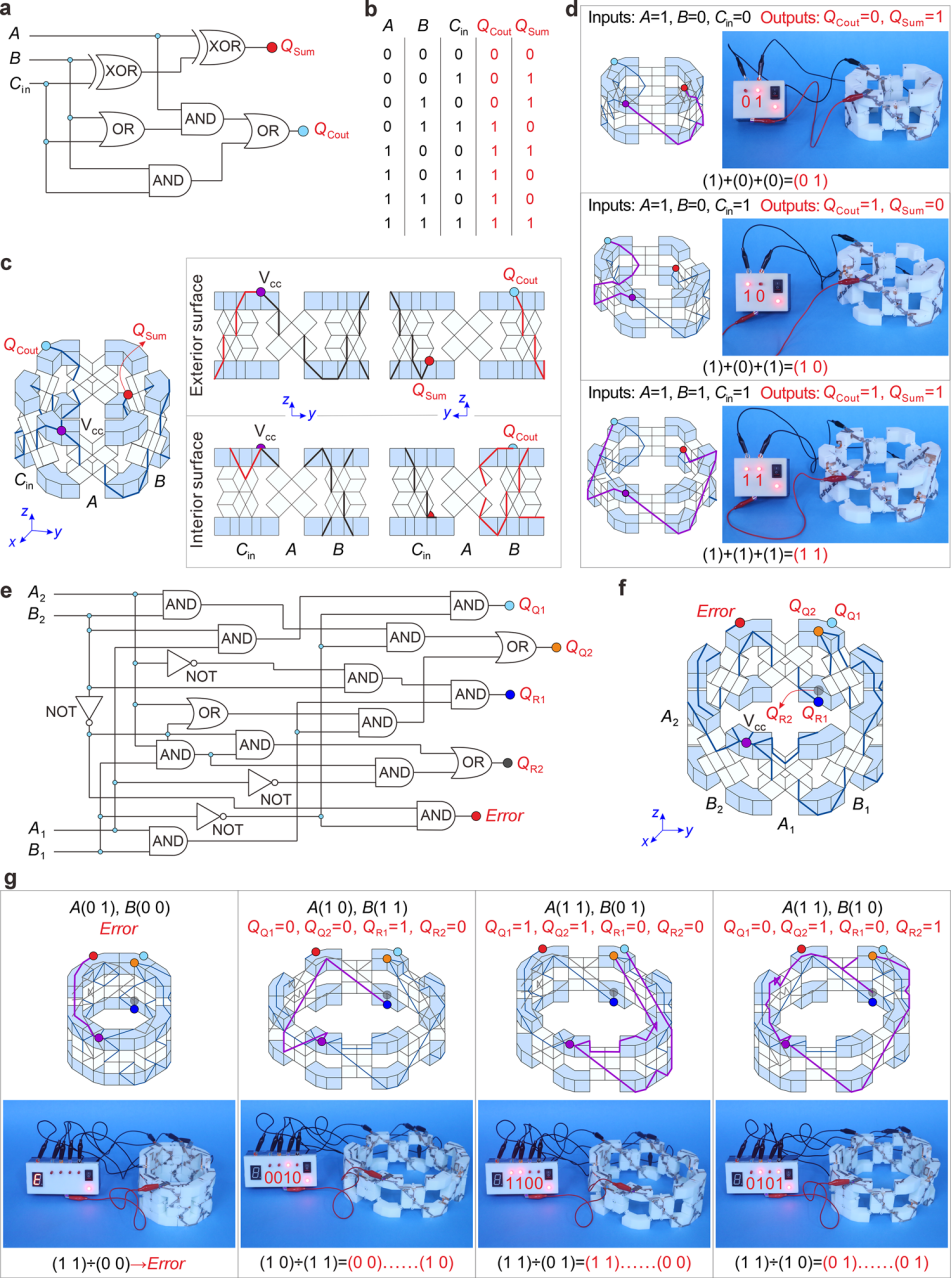
Arithmetic operators implemented on polygonal modules. a) Logic diagram of the full adder with mechanical inputs (*A*, *B*, and *C*_in_) represented in black and electrical outputs *Q*_Cout_ and *Q*_Sum_ represented by blue and red nodes, respectively. b) The truth table of the full adder. c) Schematic of the full adder on a hexagonal module with mechanical inputs (*A*, *B*, and *C*_in_); the power input terminal is the purple node, V_cc_. d) The experimental outputs of 3 possible configurations of the full adder, including the experimental prototypes and schematics. e) Logic diagram of the 2‐bit divider with mechanical inputs (*A*_1_, *A*_2_, *B*_1_, and *B*_2_) and electrical outputs (*Error*, *Q*_Q1_, *Q*_Q2_, *Q*_R1_, and *Q*_R2_). The output *Error* indicates a mathematical error when 0 is used as the divisor. f) Schematic of the 2‐bit divider on an octagonal module. g) Schematics and experimental results of four possible configurations of the 2‐bit divider. The connected paths that result in an output of 1 are highlighted in purple, whereas lines that are not connected and are obscured from view are represented by thin grey lines.

In addition to logic gates, polygonal modules can perform combinatorial logic operations such as arithmetic operations. We develop an integrated design strategy that minimizes the number of switches required for these operations and integrates them into a single module with the possibly fewest units. A single quadrilateral module can be implemented as a half adder and subtractor, as shown in Figure S7, Movie S2 (Supporting Information). For the full adder and subtractor, which requires three inputs, two quadrilateral modules have to be assembled, containing eight units (Figures S8 and S9, Supporting Information). To minimize the unit number, a hexagonal module with three inputs is perfect for a full adder, as polygonal modules with six sides can provide 3 decoupled mechanical inputs. The full adder performs the addition of three 1‐bit operands *A*, *B*, and *C*_in_ (a carry bit typically used in a parallel binary adder circuit) and yields two electrical outputs, *Q*_Sum_ (red node) and *Q*_Cout_ (light blue node), which correspond to the least significant and most significant bits of the binary output (*Q*_Cout_ *Q*_Sum_) (**Figure** **3**a). The SSoP functions for each output are extracted from the truth table in Figure 3b, which is the sum of the minterms.^[^ ^44^ ^]^ Each minterm corresponds to the product of all input Boolean terms that result in an output of 1. Here, each Boolean term within the SSoP functions corresponds to a switch in the circuit,^[^ ^45^ ^]^ so minimizing this function reduces the number of switches. The QMSoP function is an existing simplified form of the SSoP function.^[^ ^23^ ^]^ We further propose a PCSoP function by introducing the concept of temporal parallelism from PC,^[^ ^41^, ^42^ ^]^ which simplifies the QMSoP function through extracting duplicate mechanical logic network components as a single sub‐task using the common factor method (see Section S4, Supporting Information). The resulting PCSoP functions for the full adder outputs are *Q*_Cout_ = *A*(*B* + *C*_in_) + *BC*_in_ and *Q*_Sum_ = $\;A\left(\bar{B}\bar{C_{\mathrm{in}}}+BC_{\mathrm{in}}\right)+\bar{A}\left(B\bar{C_{\mathrm{in}}}+\bar{B}C_{\mathrm{in}}\right)$, which utilize only buffer (ordinary term) and NOT (inverted term, denoted by (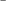)) gates as fundamental switches. Considering that the multiplication and addition operations indicate series and parallel connections between two switches, respectively, *Q*_Cout_ is formed by the parallel connection of two series combinations *A*(*B* + *C*_in_) and *BC*_in_, where *A*(*B* + *C*_in_) is a series connection between an *A*‐input buffer gate and a parallel connection of *B*‐input and *C*_in_‐input buffer gates, whose circuits are presented as red lines in Figure 3c. Similarly, the output *Q*_Sum_ is assembled using 5 buffer gates and 5 NOT gates, as indicated by the black lines on the module surface (Figure 3c). The corresponding prototype and experiments are shown in Figure 3d and Movie S3 (Supporting Information), respectively, where the hexagonal module is folded into different extreme configurations according to the inputs (*A*, *B*, *C*), and the experimental outputs of the full adder are consistent with its truth table (Figure 3b).

The full adder proposed here consists of 15 switches, which is less than the number of switches included in full adders developed using the SSoP functions (24 switches) and QMSoP functions (18 switches).^[^ ^23^ ^]^ Moreover, by leveraging the proposed universal PCSoP functions, we developed a design framework for universal combinatorial logic operations, and its automated implementation method was programmed in MATLAB (see Section S6, Supporting Information).

Moreover, owing to the four decoupled mechanical inputs, an octagonal module can be used to design all four 2‐bit arithmetic operators, with the divider implemented for the first time. Figures S11–S14 (Supporting Information) represent the three 2‐bit arithmetic operations of adding, subtracting, and multiplying the 2‐bit operands *A*(*A*_1_ *A*_2_)_2_ and *B*(*B*_1_ *B*_2_)_2_, all of which require 22 switches, with the corresponding binary outputs *Q*_Add_(*Q*_Cout_ *Q*_S1_ *Q*_S2_)_2_, *Q*_Sub_(*Q*_Bout_ *Q*_D1_ *Q*_D2_)_2_, and *Q*_Mul_ (*Q*_P1_ *Q*_P2_ *Q*_P3_ *Q*_P4_)_2_ (see Section S5, Supporting Information). The prototype and test of a 2‐bit multiplier are shown in Figure S13d and Movie S4 (Supporting Information). Notably, the implementation of 2‐bit division presents unique challenges with a more complex and irregular logic diagram (Figure 3e). To address indivisibility in division, we introduce a remainder‐preserving scheme with *Q*_Rema_(*Q*_R1_ *Q*_R2_)_2_; for divide‐by‐zero cases, an error‐handling protocol triggers the output *Error* when the divisor *B*(*B*_1_ *B*_2_)_2_ = (0 0). Figure 3f shows the 2‐bit divider implemented on an 8‐unit octagonal module, which performs the division between two operands *A*(*A*_1_ *A*_2_)_2_ and *B*(*B*_1_ *B*_2_)_2_, yielding the binary quotient *Q*_Quot_(*Q*_Q1_ *Q*_Q2_)_2_ and remainder *Q*_Rema_(*Q*_R1_ *Q*_R2_)_2_. According to the truth table in Figure S14a (Supporting Information), the PCSoP functions of the 2‐bit divider for each output can be derived as *Q*_Q1_ = $\;A_{1}\bar{B_{1}}B_{2}$, *Q*_Q2_ = $\;A_{1}B_{1}\left(A_{2}+\bar{B_{2}}\right)+\bar{B_{1}}A_{2}B_{2}$, *Q*_R1_ = $\;A_{1}B_{1}\bar{A_{2}}B_{2}$, *Q*_R2_ = $\;\bar{A_{1}}B_{1}A_{2}+B_{1}A_{2}\bar{B_{2}}$, and *Error* = $\;\bar{B_{1}}\;\bar{B_{2}}$. There are a total of 14 buffer gates and 8 NOT gates in the 2‐bit divider shown in Figure 3f and Figure S14 (Supporting Information). The corresponding prototype and experimental results are depicted in Figure 3g and Movie S5 (Supporting Information), and the outputs match the truth table (Figure S14a, Supporting Information). Thus, we demonstrated the brevity and high computational utility of polygonal modules developed using the PCSoP function.

### 2.4 Information Display and Recognition

**Figure 4:**
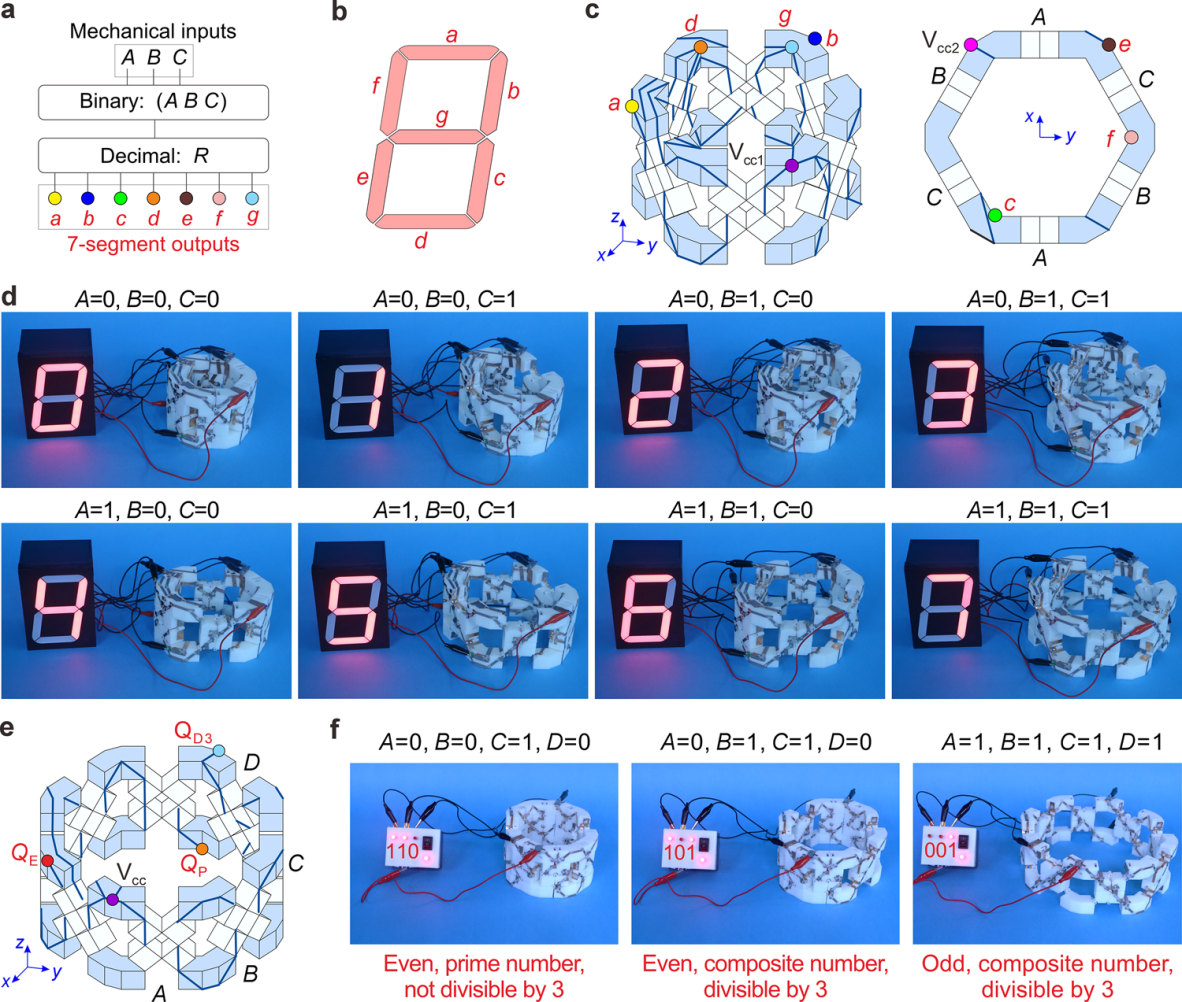
Information display and recognition functions implemented on polygonal modules. a‐d) Conceptual diagram, illustration of the 7‐segment display, design schematic, and experimental results of the hexagonal module‐based binary‐coded decimal (BCD) to 7‐segment display decoder. The BCD to 7‐segment display decoder takes three mechanical inputs *A*, *B*, and *C* and provides seven outputs *a*‐*g*, represented by the yellow, blue, green, orange, brown, pink, and light blue nodes, respectively, with each output controlling a segment of the LED display in (b). There are two power input terminals, V_cc1_ and V_cc2_, represented by the purple and magenta nodes, which are positioned at opposite ends of the hexagonal module in (c). The BCD to 7‐segment display decoder in (d) sequentially demonstrates the digits 0‐7 using the 7‐segment display. e, f) The design schematic and experimental results of the recognition module for binary‐coded decimal numbers 2‐15 implemented on the octagonal module. The recognition module deduces the decimal value from the binary operands (*A B C D*) formed by the related four mechanical inputs and assesses its mathematical properties: even (*Q*_E_ = 1) or odd (*Q*_E_ = 0), prime (*Q*_P_ = 1) or composite (*Q*_P_ = 0), and divisible by 3 (*Q*_D3_ = 1) or not divisible by 3 (*Q*_D3_ = 0). The outputs *Q*_E_, *Q*_P_, and *Q*_D3_ for each property are represented by the red, orange, and light blue nodes, respectively.

In addition to mechanical logic and computing, polygonal modules can facilitate diverse applications, such as displaying and recognizing information. To display the decimal numbers 0‐7 on a 7‐segment display, the corresponding binary numbers can be decoded by the hexagonal module with three inputs. Specifically, the hexagonal module serves as a binary‐coded decimal (BCD) to 7‐segment display decoder, which converts a binary number (*A B C*) into a decimal number *R* (0‐7) and provides electrical outputs *a*‐*g* to drive 7‐segment LEDs to display the corresponding *R* (**Figure** **4**a, b). According to the logic diagram and truth table in Figures S15a,b (Supporting Information), the PCSoP functions for outputs *a*‐*g* can be obtained. Leveraging the PCSoP functions, the designed display decoder (Figure 4c) is composed of 35 switches on a 6‐unit hexagonal module, with the seven outputs *a*‐*g* distinguished by different color nodes (Figure S15, Supporting Information). The results of the BCD to 7‐segment display decoder demonstrate that, given the appropriate inputs, the decoder can display the corresponding outputs (Figure 4d; Movie S6, Supporting Information).

Furthermore, we designed a recognition module that can simultaneously evaluate three common mathematical properties of the generated decimal numbers (Figure 4e). Utilizing the PCSoP functions, our recognition module, implemented using 25 switches on an 8‐unit octagonal module, decodes the binary number (*A B C D*) into a decimal number *R* and analyses its mathematical properties, providing three outputs: even (*Q*_E_ = 1) or odd (*Q*_E_ = 0), prime (*Q*_P_ = 1) or composite (*Q*_P_ = 0), and divisible by 3 (*Q*_D3_ = 1) or not divisible by 3 (*Q*_D3_ = 0). Since 0 and 1 are neither prime nor composite, the recognition module can take inputs in the range of 2‐15. Experiments demonstrate that this recognition module accurately assesses the mathematical properties corresponding to binary inputs 2‐15 (Figure 4f; Figure S16, and Movie S7, Supporting Information). Other properties with known input‐output relationships can also be analyzed by constructing the corresponding recognition modules.

## 3 Discussion

In summary, we proposed a set of kinematically bifurcated polygonal modules, combining with electrical circuits to construct metamaterials for integrated mechanical computing. All seven basic logic gates were integrated on a single quadrilateral module. Such a computational metamaterial enables advanced functions, including all four types of arithmetic operations (a 2‐bit divider is implemented for the first time), information display and recognition, all realized on a single polygonal module by minimizing Boolean functions and utilizing all possible module configurations. This approach significantly reduces the number of switches and units required, achieving an unprecedented level of system integration in mechanical computing.

**Figure 5:**
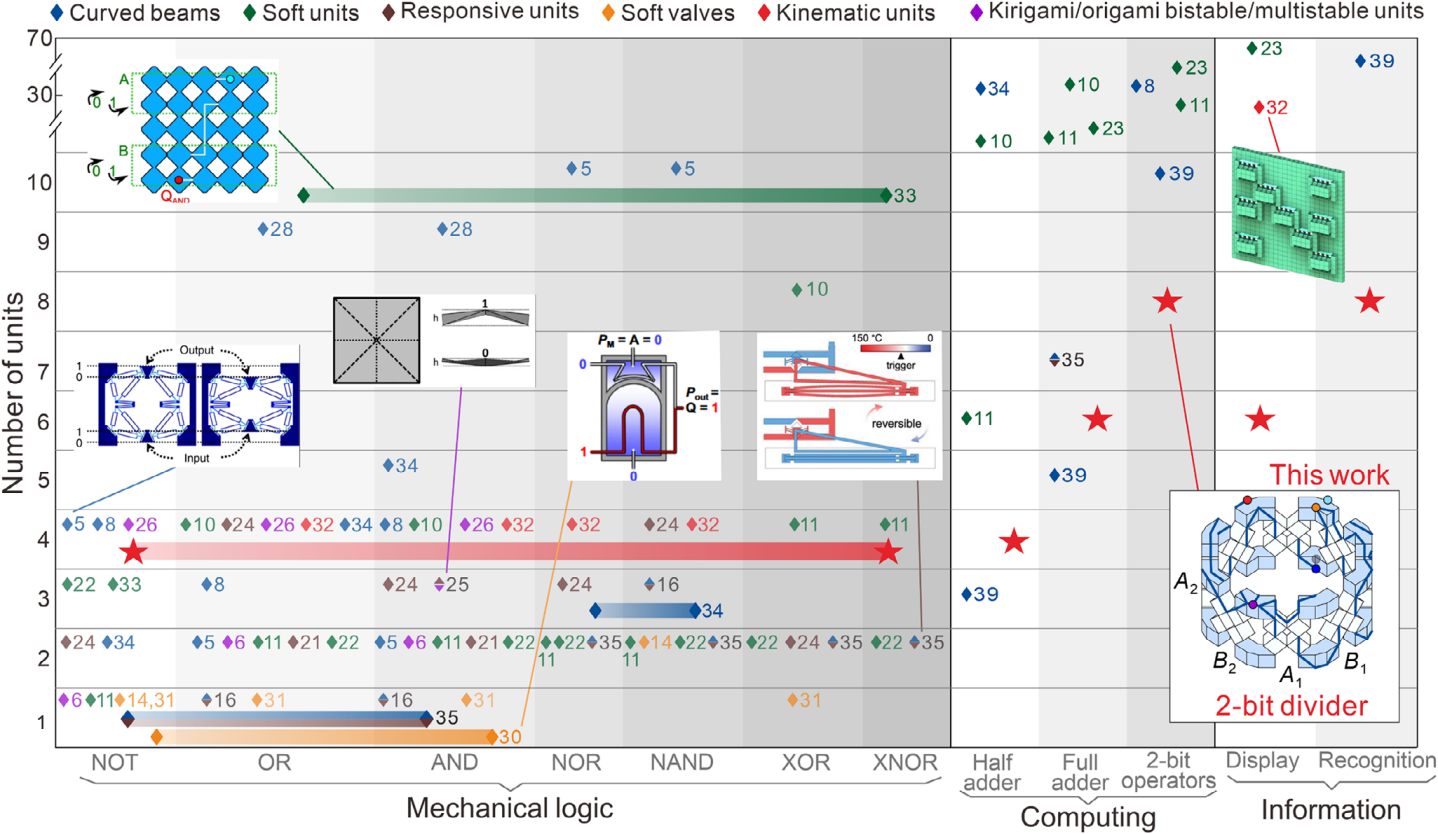
Comparison of the number of units used in mechanical logic, mechanical computing, information display and information recognition applications between our work and prior studies.^[^ ^5^, ^6^, ^8^, ^10^, ^11^, ^14^, ^16^, ^21^, ^22^, ^23^, ^24^, ^25^, ^26^, ^28^, ^30^, ^31^, ^32^, ^33^, ^34^, ^35^, ^39^ ^]^ The present work is marked by red stars, while prior works are categorized by their basic units and represented by diamonds of different colors. A representative diagram is given for each category, curved beams^[^ ^5^ ^]^ (http://creativecommons.org/licenses/by/4.0/), soft units^[^ ^33^ ^]^ (http://creativecommons.org/licenses/ by/4.0/), responsive units^[^ ^35^ ^]^ (copyright 2024, The Authors, published by Wiley‐VCH GmbH), soft valves^[^ ^30^ ^]^ (Copyright 2019, National Academy of Sciences), kinematic units^[^ ^32^ ^]^ (https://creativecommons.org/ licenses/by/4.0/), kirigami/origami bistable/multi‐stable units^[^ ^25^ ^]^ (https://creativecommons.org/licenses/by‐nc‐nd/4.0/). Diamonds with 30% transparency represent the implementation of each logic gate with its distinct structure. For multiple logic gates with the same structure, the corresponding diamond spans the interval corresponding to these gates and is shaded to indicate the spanning process.

We next compared our work with existing efforts from mechanical logic, to mechanical computing, information display and information recognition applications. Regarding logic gates, different units or structures were typically used for different gates in prior works (**Figure** **5**; Table S8 (Supporting Information); see details in Section S7, Supporting Information). Most existing work fulfil the different logic gates by different combinations of the basic buffer and NOT gates, while there are only a few exceptions, including three logic gates, NOT, OR, and AND implemented sequentially on the same mechanical transistor via a thermal reconfiguration strategy,^[^ ^27^ ^]^ NOR and NAND gates switched on a 3‐unit structure by tuning the magnitude of the applied excitation,^[^ ^12^ ^]^ and six dual‐input logic gates developed sequentially on a 10‐unit platform with different circuit arrangements.^[^ ^18^ ^]^ In contrast, our approach requires only a 4‐unit quadrilateral module to simultaneously implement all seven basic gates, which requires much fewer units than the other works.

Meanwhile, for complex mechanical computing systems that require numerous units, streamlining the system to reduce redundant units can further improve the computational utility and, consequently, the level of integration. The bottom‐up design approach, while suitable for constructing a universal design framework, typically requires a large number of units. For example, 2‐bit arithmetic operators (i.e., the adder, subtractor, and multiplier) can be implemented using up to 44 units (Figure 5).^[^ ^23^ ^]^ In contrast, through an ingenious top‐down design, half, full, and 2‐bit adders were implemented with a relatively compact system using up to 10 units.^[^ ^39^ ^]^ However, due to the lack of a generic design strategy, this approach has not yet been extended to the 2‐bit multiplication and other advanced arithmetic operations. Our approach, leveraging the proposed universal PCSoP functions and various configurations of the kinematically bifurcated, single‐DOF, polygonal modules, implements 2‐bit arithmetic operators (addition, subtraction, multiplication, and even division) on an 8‐unit octagonal module and realizes information display or recognition functions on the single polygonal module. Therefore, our polygonal module‐based logical operators, with fewer units, match or exceed the functionality of existing designs, providing an extendable design framework.

For future work, the designed integrated logic metamaterials may be extended to decagonal and larger modules, as well as module tessellations. Given an input‐output relationship for a desired function, the corresponding logic metamaterial can be achieved by arranging fundamental buffer and NOT gates under the guidance of the proposed PSCoP functions. This approach could enable more complex or customized logic operations based on polygonal modules, such as information encryption and robotic control with decision‐making. However, similar to the existing computational metamaterials, a large number of units bring a big challenge in actuating the inputs precisely, especially for the miniature system with multi‐material 3D printing or micro‐ and nano‐electromechanical systems.^[^ ^46^, ^47^, ^48^ ^]^ This necessitates further simplification and integration of the logic module, which in turn stimulates research into the innovative design of kinematic metamaterials to overcome such limitations without sole reliance on manufacturing solutions. Moreover, while our current work focuses on demonstrating mechanical computing principles through manual actuation, we are considering integrating non‐conventional actuation methods, such as stimuli‐responsive materials, to drive hinges in future work. This could further enhance the functionality and usability of mechanical computing systems.

In addition, compared with electronic computing systems, mechanical computing systems still have common problems, such as more complex operations and the need for users to identify structural deformation states to obtain some input or output information.^[^ ^11^, ^33^ ^]^ Despite these challenges, mechanical computing complements traditional electronic computing by enhancing adaptability in diverse environments, including those with extreme temperatures and radiation.^[^ ^35^, ^49^, ^50^ ^]^ Moving forward, we aim to collaborate with other scholars in this field to overcome these challenges and advance the development of mechanical computing systems.

## 4 Experimental Section

### Manufacturing the Logic Material

The manufacturing process of the logic material consists of two main steps. Initially, the basic mechanical module is constructed. The quadrilateral module (Figure S17, Supporting Information) comprises 16 cubes and 8 rectangular blocks, with *a* = 25 mm. Joints between the blocks are formed using shafts (radius *r* = 1 mm, length 9 mm) and holes (outer radius *R*_1_ = 2.5 mm, length 9 mm), with staggered arrangements and removal of material (radius *R*_2_ = 4 mm) from the cubes to avoid physical interference. Every 4 cubes can form a 4‐bar linkage, where the cubes of the 4‐bar linkage located on the front and back of the module have a thickness of *t* = 5 mm removed from the inside surface. Corresponding parts of the rectangular blocks are similarly modified to form short L‐shaped structures. A 3 × 13 × 15mm slot is created 2mm from the surface of each rectangular block to accommodate circular magnets (12 mm diameter, 3 mm thickness). The blocks were 3D‐printed using photosensitive resin by Datong Naxiao Technology Co., Ltd., while the shafts were manufactured from 304 stainless steel by Dongguan Dingyixin Metal Products Co., Ltd. Circular magnets (JD.com, Inc.) were placed in the slots during assembly. The hexagonal and octagonal modules (Figures 1d; S2, Supporting Information) are similar but replace the rectangular blocks with corner blocks consisting of two cubes and a triangular prism (angles of 60° and 45°), without requiring material removal for thickness *t*.

Next, the electrical circuit is laid out using double‐sided conductive copper strips (0.066 mm thick, Beijing Yazhong Trading Co., Ltd) glued to the model surface. Three connection types are implemented (Figure S18, Supporting Information): persistent (always connected), adjacent (activated by specific inputs, e.g., buffer gate), and diagonal (requires an intermediate block, exemplified by buffer gate). Input/output nodes are marked with copper plates and stickers. Conductive silver adhesive (Hubei Night Birch Trading Co., Ltd) is used to enhance conductivity at critical points.

### Experimental Setup

The logic operators (e.g., integrated logic module, half adder, full adder, 2‐bit multiplier, 2‐bit divider, BCD to 7‐segment display decoder, recognition module) in this work have been experimentally examined to validate the outputs with their corresponding truth tables. A test box was first designed for the logic quadrilateral module integrating seven gates, using LED states to indicate experimental outputs (1 = on, 0 = off), as illustrated in Figure S6 (Supporting Information). The box was made from 4 mm acrylic sheets, with a 4 mm panel printed by photosensitive resin containing holes for LED installation. It was powered by a 3V supply (2 AA batteries) and connected to the logic module via enameled wires (circuit diagram in Figure S19a, Supporting Information). The quadrilateral module was reconfigured to each extreme configuration according to the inputs, allowing examination of all possible states and observation of the corresponding outputs (Movie S1, Supporting Information). Similar test boxes were made for other modules, such as the half adder (circuit diagrams in Figure S19b, Supporting Information). For the 2‐bit divider, if the divisor *B*(*B*_1_ *B*_2_)_2_ = (0, 0), the error‐handling protocol triggers the output *Error*, displayed as the character ‘*E*’ on a 1‐bit, 7‐segment display (1‐inch, Jiangsu Yongning Electronic Technology Co., Ltd.). Similarly, the output of the BCD to 7‐segment display decoder is illustrated on a 3‐inch 1‐bit 7‐segment display powered by a 5V supply (Movie S7, Supporting Information).

## Conflict of Interest

The authors declare no conflict of interest.

## Author Contributions

K.X. and X.Z. created the kinematic modules. K.X. performed mechanical logic design and analysis. K.X. and J.W. performed experiments. J.M. and C.C. discussed the results. Y.C., X.Z. and K.X. wrote the manuscript. Y.C. and Z.Y. supervised the research. All the authors revised the paper.

## Supporting information



Supporting Information

Supplemental Movie 1

Supplemental Movie 2

Supplemental Movie 3

Supplemental Movie 4

Supplemental Movie 5

Supplemental Movie 6

Supplemental Movie 7

## Data Availability

The data that support the findings of this study are available in the Supporting Information of this article.
