# Supplementary material for: A kinematically Bifurcated Metamaterial for Integrated Logic Operation and Computing
Source: Adv Sci (Weinh). 2025 Jul 17;12(41):e09829. doi: 10.1002/advs.202509829 (PMC12591120; doi:10.1002/advs.202509829)
Supplement: Supplementary file 1 — Supporting Information [file ADVS-12-e09829-s007.docx]

Supporting Information

A kinematically bifurcated metamaterial for integrated logic operation and computing

Kaili Xi, Jingsong Wei, Xiao Zhang*, Jiayao Ma, Zhong You, Changqing Chen, Yan Chen*

**This PDF file includes:**

S1. Kinematic analysis of the polygonal modules

S2. Buffer and NOT gates

S3. Design of a single quadrilateral module with 7 basic logic gates

S4. Parallel computing sum of product (PCSoP) function

S5. Arithmetic operators on polygonal modules

S6. The PCSoP-based automated implementation method functions for an arbitrary logic operator in MATLAB

S7. The comparison of the number of units used in mechanical logic, computing, information display, and recognition between our work and existing ones

S8. Figures S1 to S19

S9. Tables S1 to S8

S10. Legends for Movies S1 to S7

References

**S1. Kinematic analysis of the polygonal modules**

The unit of the polygonal module in Figure S1a is a closed loop of six blocks sharing edges. Here, the blue top and bottom blocks are identical, and each is made of two cubes with a side length *a* sharing an edge and connected by a triangular prism. The two blocks are connected by two limbs and each limb is composed of two jointed pink cubes with side length *a*. Each limb has a set of three parallel hinges, and the two sets of hinges with a certain angle in the range (0, π) form a Sarrus linkage with single DOF, where the two blue blocks can move in the vertical direction. To conduct the kinematic analysis, the schematic diagram of the Sarrus linkage with Denavit and Hartenberg (D-H) notations ^[49]^ is constructed, as shown in Figure S1a. Here, *α_i_*_(_*_i_*_+1)_ (*i* = 1, 2, …, 6; when *i* + 1 > 6, *i* + 1 = 1) represents the twist angles measured from *z_i_* to *z_i_*_+1_ along the positive direction of *x_i_*_+1_, *a_i_*_(_*_i_*_+1)_ represents the link lengths between axes *z_i_* and *z_i_*_+1_, and *θ_i_* represents the rotation angles of axis *z_i_* measured from *x_i_* to *x_i_*_+1_. For the Sarrus linkage in an *N*-sided closed-loop polygonal module, the geometrical parameters satisfy

$\begin{matrix} a_{12}=a_{23}=a_{45}=a_{56}=\sqrt{2}a, a_{34}=a_{61}=0 \\ \alpha_{12}=\alpha_{23}=\alpha_{45}=\alpha_{56}=0, \alpha_{34}=\alpha_{61}=2\pi/N \end{matrix}$ , (S1)

where *N* > 2 and *a* is the side length of the pink cubes. Based on the kinematic matrix method ^[49]^, the closure equation of the Sarrus linkage is

$T_{21}T_{32}T_{43}T_{54}T_{65}T_{16}=I_{4}$, (S2)

in which the transformation matrix ***T***_(_*_i_*_+1)_*_i_* transforms between the *i*th coordinate system and the (*i*+1)th coordinate system,

$T_{\left(i+1\right)i}=\left[\begin{matrix} \cos\theta_{i} & -\cos\alpha_{i\left(i+1\right)}\sin\theta_{i} & \sin\alpha_{i\left(i+1\right)}\sin\theta_{i} & a_{i\left(i+1\right)}\cos\theta_{i} \\ \sin\theta_{i} & \cos\alpha_{i\left(i+1\right)}\cos\theta_{i} & -\sin\alpha_{i\left(i+1\right)}\cos\theta_{i} & a_{i\left(i+1\right)}\sin\theta_{i} \\ 0 & \sin\alpha_{i\left(i+1\right)} & \cos\alpha_{i\left(i+1\right)} & 0 \\ 0 & 0 & 0 & 1 \end{matrix}\right]$. (S3)

Two sets of solutions for the kinematic angle *θ_i_* can be obtained from Eqs. (S1) to (S3).

Path I:

$\theta_{1}=\frac{-\theta_{2}}{2},\theta_{3}=\text{π+}\theta_{1},\theta_{4}=\theta_{1},\theta_{5}=\theta_{2},\theta_{6}=\theta_{4}-\text{π}$. (S4)

Path II:

$\theta_{1}=\text{π}-\frac{\theta_{2}}{2},\theta_{3}=\theta_{1}-\text{π},\theta_{4}=-\theta_{1},\theta_{5}=-\theta_{2},\theta_{6}=\theta_{4}-\text{π}$. (S5)

As shown in Figure S1a, the dihedral angles *θ*_Α_ = π/2 + *θ*_2_ and *θ*_B_ = π/2 + *θ*_5_ are in the range [0, π], whereas Figure 1a shows two motion paths related to the relationships between *θ*_Α_ and *θ*_B_. Along path I (the blue line), *θ*_Α_ = *θ*_B_, the two pairs of pink cubes are always symmetrically distributed during motion, whereas they are asymmetrically distributed along path II (the orange line), as *θ*_Α_ = π - *θ*_B_. The two motion paths can be switched at the bifurcation point with *θ*_Α_ = *θ*_B_ = π/2.

Through a 4-bar linkage formed by two pink-cube pairs, two Sarrus linkages can be formed in a one-DOF assembly. Therefore, *N* Sarrus linkages can form an *N*-sided closed-loop polygonal module through *N* 4-bar linkages (*N* > 2), as shown in Figure S1b-d, with *N* = 3, 4, 5, and 6, respectively. Due to the high symmetry, the Sarrus linkages and 4-bar linkages move synchronously, which indicates that the modules still have a single DOF. When the modules are in the extreme configurations with *θ_i_* = 0 or π, the length of each side of the closed-loop polygonal module can be only 2 or 4 times the side length of pink cubes, i.e., 2*a* or 4*a*. Thus, in the top-view plane, the vector ***L****_i_* representing each side can be expressed as

$L_{i}=2x_{i}a\left[\begin{matrix} \cos\left(2\text{π}\left(i-1\right)/N\right) & \sin\left(2\text{π}\left(i-1\right)/N\right) \end{matrix}\right]^{\text{T}}$, (S6)

where *x_i_*=1 or 2 is the coefficient of the side length corresponding to *θ_i_*=0 or π. For the *N*-sided closed-loop polygons, we have

$L_{1}+L_{2}+\ldots+L_{N}=0$. (S7)

Combining Eqs. (S6) and (S7), we employed an exhaustive search in MATLAB to identify all potential solutions. If *N* is a prime number, the solutions are constrained to all *x_i_* = 1 or 2 for *i* = 1, 2, ..., *N*, as depicted in Figure S1b for the triangular and pentagonal modules, implying the absence of kinematic bifurcations. When *N* is a composite number, i.e., *N* = *m* × *n*, multiple solutions arise. These can be obtained by dividing the sides of the polygonal module into *m* identical groups based on each factor *n* (excluding *n* = 1 and *N*). For the group with factor *n* representing the number of decoupled inputs, 2*^n^* possible solutions can be obtained by permuting the side lengths of 2*a* or 4*a*. For instance, when *N* = 4 = 2 × 2, which has a factor *n* of 2, the coefficients *x*_1_, *x*_2_, *x*_3_, and *x*_4_ of the length of each side can be divided into two identical groups: *x*_1_ and *x*_2_ in one group, and *x*_3_ and *x*_4_ in the other. Through permutations and combinations of *x_i_* = 1 or 2 for one of these groups, 2^2^ (=$C_{2}^{0}+C_{2}^{1}+C_{2}^{2}$) sets of solutions for the coefficients of the lengths of each side can be obtained as

$x_{1}=x_{2}=x_{3}=x_{4}=1$, (S8a)

$x_{1}=x_{3}=1,x_{2}=x_{4}=2$, (S8b)

$x_{1}=x_{3}=2,x_{2}=x_{4}=1$, (S8c)

$x_{1}=x_{2}=x_{3}=x_{4}=2$. (S8d)

The corresponding four extreme configurations of the quadrilateral module, which can be switched through the kinematic bifurcation point at *θ*_Α_ = *θ*_B_ = π/2, are shown in Figure S1c. These extreme configurations involve two decoupled inputs, *θ*_Α_ and *θ*_B_. It should be noted that the blue corner blocks (Figure S1a) are replaced with rectangular blocks to reduce the amount of material, yielding a special Sarrus linkage that forms the quadrilateral module (Figure 1c and Figure S1c). The hexagonal module with *N* = 6 = 3 × 2 has two factors, 3 and 2. If we take factor 3 as the number of decoupled inputs, *θ*_Α_, *θ*_B_, and *θ*_C_, there are 2^3^ (=$C_{3}^{0}+C_{3}^{1}+C_{3}^{2}+C_{3}^{3}$) extreme configurations. Alternatively, if we take factor 2 as the number of decoupled inputs, *θ*_Α1_ and *θ*_B1_, there are 2^2^ extreme configurations (Figure S1d). Thus, more extreme configurations can be obtained in the case of three decoupled inputs. The octagonal module with *N* = 8 = 4 × 2 has 2^4^ extreme configurations in the case of four decoupled inputs, *θ*_Α_, *θ*_B_, *θ*_C_, and *θ*_D_. If we consider the case of only two decoupled inputs, the corresponding 2^2^ extreme configurations overlap with the 2^4^ extreme configurations in the case of four decoupled inputs, as shown in Figure S2. Specifically, for closed-loop polygonal modules with an even number of sides, *m* = 2 is preferred to maximize *n*, thereby obtaining the maximum number of decoupled inputs from the same polygonal modules and achieving the maximum 2*^n^* extreme configurations.

By removing the triangular prism connecting the adjacent cubes in the blue blocks, the unit of the polygonal module, a closed loop of six blocks, will be transformed from a Sarrus linkage (Figure S1a) to a planar 6-bar linkage with six parallel hinges (Figure S3a). According to the schematic diagram of the planar 6-bar linkage with D-H notations in Figure S3a, Eq. (S1) can be modified as follows:

$\begin{matrix} a_{12}=a_{23}=a_{45}=a_{56}=\sqrt{2}a, a_{34}=a_{61}=2a \\ \alpha_{12}=\alpha_{23}= \alpha_{34}=\alpha_{45}=\alpha_{56}=\alpha_{61}=0 \end{matrix}$ , (S9)

Based on Eq. (S9), (S2), and (S3), it can be concluded that the kinematic angle *θ_i_* satisfies the following relationship:

$\theta_{2}=-2\theta_{3},\text{ }\theta_{5}=-2\theta_{6},\text{ }\theta_{1}-\theta_{3}+\theta_{4}-\theta_{6}=0$ , (S10)

Therefore, when three kinematic angles are determined, all the kinematic angles or the configuration of the mechanism can be determined; that is, the planar 6-bar linkage has three DOFs, making it more difficult to fold into the target configuration. As shown in Figure S3b, a planar straight-line metamaterial can be constructed by arranging three planar 6-bar linkages along a straight line and assembling them through 4-bar linkages formed by two pink-cube pairs from adjacent planar 6-bar linkages. Through a similar kinematic analysis, it is found that this planar straight-line metamaterial has seven DOFs, leading to asynchronous motion. Specifically, the pink cubes of the planar 4-bar linkage on the left side are tightly fitted, while the cubes on the right remain open, which means that the motion of those linkages is independent. In addition, Figure S3c provides another straight-line metamaterial formed by two Sarrus linkages with a planar 6-bar linkage between them. Compared to the planar straight-line metamaterial composed entirely of 6-bar planar linkages (Figure S3b), this metamaterial has fewer degrees of freedom (three DOFs), yet it still moves asynchronously. These two types of straight-line metamaterials are not suitable for mechanical computations because their motion paths are uncertain. In detail, when one 4-bar linkage reaches a tight-fitting configuration (with pink cubes fitted tightly), which can be abstracted as the mechanical bit state 0 or 1, the other units do not reach a similar configuration simultaneously. Instead, they may remain in an undefined, non-tight-fitting, random configuration.

Furthermore, a single-DOF straight-line metamaterial (Figure S3d) can be constructed by assembling four Sarrus linkages, with the middle two merged by sharing side surfaces (along the white lines). However, while this straight-line metamaterial can only abstract two mechanical bits due to its two 4-bar linkages, arranging the same number of Sarrus linkages in a closed-loop circle to form a quadrilateral module yields four 4-bar linkages, enabling the abstraction of four mechanical bits. Additionally, the kinematic bifurcation of the Sarrus limbs' motion at the two edges of this straight-line metamaterial is uncontrolled. Thus, arranging metamaterial units in a closed-loop circle (Figure 1c) is more advantageous. The closed-loop metamaterial maintains a single DOF, enables switching between multiple motion paths, and facilitates transitions to various extreme configurations via kinematic bifurcation, while also allowing all units within the metamaterial to be used for mechanical computing.

**S2. Buffer and NOT gates**

Figure S4 presents schematics of various buffer and NOT gates used in this paper to construct dual-input logic gates and combinatorial logic operators. Specifically, Figure S4a illustrates an *A*-input buffer gate arranged vertically between two rectangular blocks, while Figure S4b and c display two types of *A*-input buffer gates oriented diagonally, and Figure S3d shows an *A*-input buffer gate arranged horizontally. These diverse buffer gates meet the different position requirements for arranging combinatorial logic circuits within polygonal modules. When *A*=1, the two cube pairs rotate outwards to fit tightly, activating the circuit with an output *Q*_Buf_ = 1. Similarly, Figure S4e and f show *B*-input buffer gates arranged vertically and diagonally, respectively. Likewise, Figures S4g-i illustrate *A*-input NOT gates arranged horizontally, vertically, and diagonally, while Figures S4j to l present *B*-input NOT gates oriented vertically, diagonally, and laterally.

**S3. Design of a single quadrilateral module with 7 basic logic gates**

In this section, we investigate the integration of a single-input gate (NOT) and six dual-input logic gates (OR, NOR, AND, NAND, XOR, and XNOR) into a single 4-unit quadrilateral module. First, we introduce the construction of the dual-input logic gates, NOR, AND, NAND, XOR, and XNOR, which are not described in the main text, using two basic single-input logic gates, buffer and NOT. The NOR gate is the inverse of the OR gate, and its logical formulation can be simplified as *Q*_NOR_=$\bar{A+B}=\bar{A}\bar{B}$ according to Demorgan’s theorems. As mentioned earlier, multiplication and addition represent series and parallel connections between two switches, respectively, so the NOR gate can be implemented by connecting two NOT gates in series (Figure S5a). The AND gate (Figure S5b) is formed with two buffer gates in series, with the input and output nodes represented by purple and green dots marked as V_cc_ and *Q*_AND_, respectively. Its logical formulation is *Q*_NOR_=*AB*, and *Q*_AND_ = 1 occurs only when *A* = *B* = 1. Similarly, the NAND gate can be formulated as *Q*_NAND_=$\bar{AB}=\bar{A}+\bar{B}$ and is composed of two NOT gates in parallel (Figure S5c). Moreover, the XOR and XNOR logic gates are constructed to distinguish the values of two inputs *A* and *B* (Figure S5d, e), with the lines on the left and back surfaces obscured by the module shown with 50% transparency. The logical formulation of the XOR gate is *Q*_XOR_=$A\bar{B}+\bar{A}B$; this gate consists of two sets of logic gates in parallel: one set is a series connection of an *A*-input buffer gate and a *B*-input NOT gate, and the other set is a series connection of a *B*-input buffer gate and an *A*-input NOT gate. Its output *Q*_XOR_ = 1 when the two inputs *A* and *B* are different. The XNOR gate, which has the logical formulation *Q*_XNOR_=$AB+\bar{A}\bar{B}$, outputs 1 when the two inputs *A* and *B* are identical (Figure S5e). It is also formed by two sets of logic gates in parallel: one set is a series connection of an *A*-input buffer gate and a B-input buffer gate, whereas the other set is a series connection of an *A*-input NOT gate and a B-input NOT gate.

Next, we demonstrate the simultaneous implementation of the aforementioned logic gates on a quadrilateral module. These seven gates comprise a total of 17 switches (8 buffer gates and 9 NOT gates). The NOT, OR, NOR, AND, and NAND logic gates (Figure 2b, c and Figure S5a-c), whose circuits are denoted in orange, navy, black, red, and blue, respectively, are integrated on the four exterior surfaces of the quadrilateral module utilizing shared surfaces and partial circuits (Figure S5g). Specifically, the NOT, OR, and NOR gates are placed on the front and right exterior surfaces. The AND gate, after a 180° rotation around the *z*-axis, is positioned on the left and rear exterior surfaces, whereas the NAND gate, after being mirrored across the *z*-*y* plane, is arranged on the right and rear exterior surfaces. The front faces of the quadrilateral module are chosen arbitrarily, and the gates are placed in sequence. The XOR and XNOR gates (Figure S5d, e) are integrated on the interior surfaces of the quadrilateral module. Examining Figure S5d and S5e, along with their logic formulas *Q*_XOR_=$A\bar{B}+\bar{A}B$ and *Q*_XNOR_=$AB+\bar{A}\bar{B}$, it is evident that the compositions of the two gates are identical. The discrepancy arises from the series-parallel arrangement of the buffer and NOT gates, which can be rectified by repositioning the input and output nodes (Figure S5h), enabling the construction of both the XOR and XNOR gates utilizing four switches on the four interior surfaces. By simply combining the overlapping lines at the same position into a single circuit, we construct a single quadrilateral module with all seven gates, as illustrated in Figure S5i. The prototype of the integrated module with seven gates was fabricated and experimentally assessed, as shown in Figure S6.

**S4. Parallel computing sum of product (PCSoP) function**

Owing to the design advantages of the polygonal module, PC ^[41,42]^ strategies can be applied to the standard sum of the product (SSoP) function simplified by the Quine–McCluskey (QM) algorithm ^[45]^ to further simplify the logic function, which is denoted as the parallel computing sum of the product (PCSoP) function. Taking the 2-bit adder on an octagonal module in Figure S11 as an example, we introduce the method of obtaining the PCSoP functions, with the automated program detailed in Section S6. The 2-bit adder is used to add the two 2-bit operands *A*(*A*_1_ *A*_2_)_2_ and *B*(*B*_1_ *B*_2_)_2_ (*A* plus *B*) and has binary outputs *Q*_Add_(*Q*_Cout_ *Q*_S1_ *Q*_S2_)_2_. The whole calculation process of PCSoP functions can be implemented through an automatic program divided into three steps.

First, the SSoP function is extracted according to the input‒output truth table in Figure S11b, which is the sum of the minterms corresponding to each output bit. Each minterm contains the product of all input Boolean terms, where if the input *A*_1_ is 0, the inverse of the input $\bar{A_{1}}$ is included in the product, and vice versa. The SSoP functions for the outputs *Q*_Cout_, *Q*_S1_, and *Q*_S2_ are

$Q_{\mathrm{Cout}}=\overline{A_{1}}A_{2}B_{1}B_{2}+A_{1}\overline{A_{2}}B_{1}\overline{B_{2}}+A_{1}\overline{A_{2}}B_{1}B_{2}+A_{1}A_{2}\overline{B_{1}}B_{2}+A_{1}A_{2}B_{1}\overline{B_{2}}+A_{1}A_{2}B_{1}B_{2}$, (S11)

$\begin{matrix} Q_{\mathrm{S}1}=\overline{A_{1}}\overline{A_{2}}B_{1}\overline{B_{2}}+\overline{A_{1}}\overline{A_{2}}B_{1}B_{2}+\overline{A_{1}}A_{2}\overline{B_{1}}B_{2}+\overline{A_{1}}A_{2}B_{1}\overline{B_{2}} \\ +A_{1}\overline{A_{2}}\overline{B_{1}}\overline{B_{2}}+A_{1}\overline{A_{2}}\overline{B_{1}}B_{2}+A_{1}A_{2}\overline{B_{1}}\overline{B_{2}}+A_{1}A_{2}B_{1}B_{2} \end{matrix}$, (S12)

$Q_{\mathrm{S}2}=\overline{A_{2}}B_{2}+A_{2}\overline{B_{2}}$. (S13)

Each Boolean term in the function corresponds to a switch in the circuit. The negated terms ($\bar{}$) correspond to NOT gates, whereas the other terms represent buffer gates. Multiplication signifies a series connection between two switches, and addition denotes a parallel connection. Therefore, the number of switches can be reduced by simplifying the SSoP functions.

Second, a canonical function minimization, the QM algorithm, is applied to the SSoP functions to obtain the QMSoP forms ^[23]^. The modified QMSoP functions for *Q*_Cout_, *Q*_S1_, and *Q*_S2_ are

$Q_{\mathrm{Cout}}=A_{2}B_{1}B_{2}+A_{1}B_{1}+A_{1}A_{2}B_{2}$, (S14)

$Q_{\mathrm{S}1}=\overline{A_{1}}\overline{A_{2}}B_{1}+\overline{A_{1}}B_{1}\overline{B_{2}}+\overline{A_{1}}A_{2}\overline{B_{1}}B_{2}+A_{1}\overline{A_{2}}\overline{B_{1}}+A_{1}\overline{B_{1}}\overline{B_{2}}+A_{1}A_{2}B_{1}B_{2}$, (S15)

$Q_{\mathrm{S}2}=\overline{A_{2}}B_{2}+A_{2}\overline{B_{2}}$. (S16)

Third, because the polygonal module enables the bifurcation of signal transmission, i.e., one input to multiple outputs, the concept of temporal parallelism in PC can be applied to the QMSoP functions, which can be reduced to the PCSoP functions through a common factor extraction method. The corresponding logical circuits of the extracted common factors can pass their outputs to multiple subsequent circuits, resulting in a reduction in the number of switches within the logical circuits. The simplified PCSoP functions for *Q*_Cout_, *Q*_S1_, and *Q*_S2_ are as follows:

$Q_{\mathrm{Cout}}=\left(A_{1}+B_{1}\right)A_{2}B_{2}+A_{1}B_{1}$, (S17)

$Q_{\mathrm{S}1}=\left(\overline{A_{1}}B_{1}+A_{1}\overline{B_{1}}\right)\left(\overline{A_{2}}+\overline{B_{2}}\right)+\left(\overline{A_{1}}\overline{B_{1}}+A_{1}B_{1}\right)A_{2}B_{2}$, (S18)

$Q_{\mathrm{S}2}=\overline{A_{2}}B_{2}+A_{2}\overline{B_{2}}$, (S19)

Here, *Q*_S1_ and *Q*_Cout_ have been substantially simplified, while *Q*_S2_ remains unchanged.

The derived PCSoP functions are subsequently used to connect various buffer and NOT gates (Section S2) in series or parallel to implement the 2-bit adder on an octagonal module. Specifically, in polynomial multiplication, such as the first term in Eq. (S18) for *Q*_S1_, $\left( \bar{A_{1}}B_{1}+A_{1}\bar{B_{1}} \right)\left( \bar{A_{2}}+\bar{B_{2}} \right)$ indicates a series connection of two parallel combinations $\bar{A_{1}}B_{1}+A_{1}\bar{B_{1}}$ and $\bar{A_{2}}+\bar{B_{2}}$. Thus, the concept of temporal PC was effectively used to minimize the number of switches in the logic system. Here, only common factors at the start and end of the circuit are allowed to be extracted; otherwise, it leads to an output error. The “start” and “end” here refer to the spatial positions of the inputs on the module. More specifically, for example, in a lengthy formula such as $A_{1}A_{2}A_{3}B_{1}B_{2}B_{3}+\bar{A_{1}}A_{2}A_{3}\bar{B_{1}}B_{2}B_{3}$, we can extract only the common factor *B*_2_*B*_3_ in simplified form $\left( A_{1}A_{2}A_{3}B_{1}+\bar{A_{1}}A_{2}A_{3}\bar{B_{1}} \right)B_{2}B_{3}$, and it is not permissible to extract a common factor such as *A*_2_*A*_3_ from the middle of the formula.

**S5. Arithmetic operators on polygonal modules**

This section introduces the arithmetic operations on polygonal modules in detail, including half/full adders/subtractors and 2-bit arithmetic operations for addition, subtraction, multiplication, and division. The design of the half adder is investigated in Figure S7a, which performs the addition (*A* plus *B*) with two 1-bit numbers *A* and *B* and provides a binary output (*Q*_Cout_ *Q*_Sum_). The half adder consists of the AND and XOR logic gates, where the output *Q*_Cout_ = AND (*A*, *B*) represents the carry bit, i.e., the circuit between the power input terminal V_cc_ (the purple node) and the output *Q*_Cout_ (the light blue node) on the outside of the module is the AND logic gate. The output *Q*_Sum_ = XOR(*A*, *B*) represents the result of the addition, and its output node is highlighted in red. The truth table of the half adder is shown in Figure S7b, and its simulation and experimental results in all four cases agree well with the truth table, see Figure S7c and Movie S2. Connected paths leading to an output of 1 are highlighted in purple, while lines not connected and obscured from the current view are represented by grey thin lines. Similarly, the half subtractor results in the subtraction (*A* minus *B*) of two 1-bit numbers *A* and *B*, and allows binary output (*Q*_Bout_ *Q*_Diff_). Here, the binary digit *Q*_Bout_ corresponds to a decimal value of -2. The power input terminal V_cc_ is represented by a green node, and the outputs *Q*_Bout_ and *Q*_Diff_ are represented by pink and orange nodes, respectively, see Figure S7d. The output *Q*_Bout_ =$\bar{A}B$ consists of a NOT gate with input *A* and a buffer gate with input *B*, resulting in a comparer (if *A* < *B*, then *Q*_Bout_ = 1; otherwise *Q*_Bout_ = 0, where *Q*_Bout_ is the borrow bit). The output *Q*_Diff_ = XOR (*A*, *B*) represents the result of the subtraction after borrowing. The truth table of the half subtractor is shown in Figure S7e, and its simulation and experimental results in all four cases are provided in Figure S7f and Movie S2.

Since the quadrilateral module can only accept two decoupled mechanical inputs, further mechanical computing must be achieved based on multiple modules in series or parallel. The schematics of the full adder and subtractor, constructed by connecting two quadrilateral modules in series, are presented in Figures S8 and S9. The full adder performs 3-bit addition (*A*_1_ plus *A*_2_ plus *B*) with 3 inputs (*A*_1_, *A*_2_, *B*) yielding two electrical outputs, *Q*_Sum_ (red node) and *Q*_Cout_ (light blue node), corresponding to the least significant and most significant bits of the binary output (*Q*_Cout_ *Q*_Sum_), see Figure S8. Combined with the logic diagram and truth table in Figures S8a, b, the PCSoP functions of outputs *Q*_Cout_ and *Q*_Sum_ are

$Q_{\text{Cout}}=A_{1}\left(A_{2}+B\right)+A_{2}B$, (S20)

$Q_{\text{Sum}}=A_{1}\left(\overline{A_{2}}\overline{B}+A_{2}B\right)+\overline{A_{1}}\left(A_{2}\overline{B}+\overline{A_{2}}B\right)$. (S21)

Specifically, the output *Q*_Cout_ represents the carry bit, and the circuit between the power input terminal V_cc_ (the purple node) and the output *Q*_Cout_ is composed of 5 buffer gates. The output *Q*_Cout_ represents the result of the addition, consisting of 5 buffer gates and 5 NOT gates, see Figure S8c. Figure S8d shows the simulation of the full adder in all eight cases.

Similarly, the full subtractor is also implemented based on two modules in series, performing the 3-bit subtraction *(A*_1_ minus *A*_2_ minus *B*) and providing two electrical outputs, *Q*_Diff_ (red node) and *Q*_Bout_ (light blue node), corresponding to the least and most significant bits of the binary output (*Q*_Bout_ *Q*_Diff_), see Figure S9. Note that the binary number *Q*_Bout_ is still equivalent to decimal -2. Combined with the logic diagram and truth table in Figures S9a, b, the PCSoP functions of outputs *Q*_Bout_ and *Q*_Diff_ are

$Q_{\text{Bout}}=\overline{A_{1}}\left(A_{2}+B\right)+A_{2}B$, (S22)

$Q_{\text{Diff}}=A_{1}\left(\overline{A_{2}}\overline{B}+A_{2}B\right)+\overline{A_{1}}\left(A_{2}\overline{B}+\overline{A_{2}}B\right)$. (S23)

Thus, the full subtractor can be obtained by replacing an *A*_1_-input buffer gate with a corresponding NOT gate, and the simulation of the full subtracter in all cases is shown in Figure S9d.

Owing to its three decoupled inputs, the hexagonal module is suitable for creating a full adder or subtractor and requires less material compared to two quadrilateral modules in series. Figure S10 illustrates the design and simulation of a full subtractor constructed on a hexagonal module, which performs 3-bit subtraction *(A* minus *B* minus *C*_in_) with 3 inputs (*A*, *B*, *C*_in_), giving two electrical outputs, *Q*_Diff_ (red node) and *Q*_Bout_ (light blue node), corresponding to the least and most significant bits of the binary output (*Q*_Bout_ *Q*_Diff_). A comparison between its logic diagram (Figure S10a) and that of the full adder (Figure 3a) shows that the output *Q*_Diff_ is congruent with the output *Q*_Sum_; the output *Q*_Bout_ can be obtained by replacing an *A*-input buffer gate in *Q*_Cout_ with the corresponding NOT gate.

Furthermore, the octagonal module with four decoupled inputs can be employed to implement the 2-bit arithmetic operators, performing the addition, subtraction, multiplication, and division between the 2-bit operators *A*(*A*_1_ *A*_2_)_2_ and *B*(*B*_1_ *B*_2_)_2_, as shown in Figures S10-13, respectively. Among these, the 2-bit adder has been detailed in the Section S4, hence, the remaining 2-bit operators will be discussed here. Figure S12 illustrates the 2-bit subtractor on an octagonal module, which carries out the subtraction between two operands *A*(*A*_1_ *A*_2_)_2_ and *B*(*B*_1_ *B*_2_)_2_ and provides the binary output *Q*_Sub_(*Q*_Bout_ *Q*_D1_ *Q*_D2_)_2_. In this context, the output *Q*_Bout_ represents the most significant bits of the binary output *Q*_Sub_(*Q*_Bout_ *Q*_D1_ *Q*_D2_)_2_, which is equal to -4 in decimal. According to the logic diagram and truth table in Figure S12a, b, the SSoP functions for the outputs *Q*_Bout_, *Q*_D1_, and *Q*_D2_ can be extracted as follows

$Q_{\text{Bout}}=\overline{A_{1}}\overline{A_{2}}\overline{B_{1}}B_{2}+\overline{A_{1}}\overline{A_{2}}B_{1}\overline{B_{2}}+\overline{A_{1}}\overline{A_{2}}B_{1}B_{2}+\overline{A_{1}}A_{2}B_{1}\overline{B_{2}}+\overline{A_{1}}A_{2}B_{1}B_{2}+A_{1}\overline{A_{2}}B_{1}B_{2}$, (S24)

$\begin{matrix} Q_{\text{D1}}=\overline{A_{1}}\overline{A_{2}}\overline{B_{1}}B_{2}+\overline{A_{1}}\overline{A_{2}}B_{1}\overline{B_{2}}+\overline{A_{1}}A_{2}B_{1}\overline{B_{2}}+\overline{A_{1}}A_{2}B_{1}B_{2} \\ +A_{1}\overline{A_{2}}\overline{B_{1}}\overline{B_{2}}+A_{1}\overline{A_{2}}B_{1}B_{2}+A_{1}A_{2}\overline{B_{1}}\overline{B_{2}}+A_{1}A_{2}\overline{B_{1}}B_{2} \end{matrix}$, (S25)

$Q_{\text{D2}}=\overline{A_{2}}B_{2}+A_{2}\overline{B_{2}}$. (S26)

Subsequently, the function minimization QM algorithm is applied to the SSoP to obtain its QMSoP forms:

$Q_{\text{Bout}}=\overline{A_{2}}B_{1}B_{2}+\overline{A_{1}}B_{1}+\overline{A_{1}}\overline{A_{2}}B_{2}$, (S27)

$Q_{\text{D1}}=\overline{A_{1}}\overline{A_{2}}\overline{B_{1}}B_{2}+\overline{A_{1}}B_{1}\overline{B_{2}}+\overline{A_{1}}A_{2}B_{1}+A_{1}\overline{B_{1}}\overline{B_{2}}+A_{1}\overline{A_{2}}B_{1}B_{2}+A_{1}A_{2}\overline{B_{1}}$, (S28)

$Q_{\text{D2}}=\overline{A_{2}}B_{2}+A_{2}\overline{B_{2}}$. (S29)

Next, these QMSoP functions can be further reduced into the PCSoP functions by a common factor extraction method. The resulting simplified PCSoP functions for outputs *Q*_Bout_, *Q*_D1_, and *Q*_D2_ are

$Q_{\text{Bout}}=\left(\overline{A_{1}}+B_{1}\right)\overline{A_{2}}B_{2}+\overline{A_{1}}B_{1}$, (S30)

$Q_{\text{D1}}=\left(\overline{A_{1}}B_{1}+A_{1}\overline{B_{1}}\right)\left(A_{2}+\overline{B_{2}}\right)+\left(A_{1}B_{1}+\overline{A_{1}}\overline{B_{1}}\right)\overline{A_{2}}B_{2}$, (S31)

$Q_{\text{D2}}=\overline{A_{2}}B_{2}+A_{2}\overline{B_{2}}$, (S32)

in which *Q*_D1_ and *Q*_Bout_ have been reduced to a great extent while *Q*_D2_ remains unchanged. Leveraging its PCSoP functions, the 2-bit subtractor designed on an octagonal module is depicted in Figure S12c, which consists of 22 switches in total. Figure S12d shows the simulation outputs of the 2-bit subtractor.

In addition, as shown in Figure S13, a 2-bit multiplier is constructed and demonstrated on an octagonal module, which performs the multiplication between two binary operands *A*(*A*_1_ *A*_2_)_2_ and *B*(*B*_1_ *B*_2_)_2_, and allows the binary output *Q*_Mul_(*Q*_P1_ *Q*_P2_ *Q*_P3_ *Q*_P4_)_2_. Combined with its logic diagram and truth table in Figure S13a, b, the SSoP functions for the outputs *Q*_P1_, *Q*_P2_, *Q*_P3_, and *Q*_P4_ are

$Q_{\text{P1}}=A_{1}A_{2}B_{1}B_{2}$, (S33)

$Q_{\text{P2}}=A_{1}\overline{A_{2}}B_{1}\overline{B_{2}}+A_{1}\overline{A_{2}}B_{1}B_{2}+A_{1}A_{2}B_{1}\overline{B_{2}}$, (S34)

$Q_{\text{P3}}=\overline{A_{1}}A_{2}B_{1}\overline{B_{2}}+\overline{A_{1}}A_{2}B_{1}B_{2}+A_{1}\overline{A_{2}}\overline{B_{1}}B_{2}+A_{1}\overline{A_{2}}B_{1}B_{2}+A_{1}A_{2}\overline{B_{1}}B_{2}+A_{1}A_{2}B_{1}\overline{B_{2}}$, (S35)

$Q_{\text{P4}}=A_{2}B_{2}$. (S36)

Next, using the same method as above, its QMSoP functions can be obtained:

$Q_{\text{P1}}=A_{1}A_{2}B_{1}B_{2}$, (S37)

$Q_{\text{P2}}=A_{1}\overline{A_{2}}B_{1}+A_{1}B_{1}\overline{B_{2}}$, (S38)

$Q_{\text{P3}}=\overline{A_{1}}A_{2}B_{1}+A_{1}\overline{A_{2}}B_{2}+A_{1}\overline{B_{1}}B_{2}+A_{2}B_{1}\overline{B_{2}}$, (S39)

$Q_{\text{P4}}=A_{2}B_{2}$. (S40)

Subsequently, the simplified PCSoP functions for outputs *Q*_P1_, *Q*_P2_, *Q*_P3_, and *Q*_P4_ can be derived from these QMSoP functions as follows:

$Q_{\text{P1}}=A_{1}A_{2}B_{1}B_{2}$, (S41)

$Q_{\text{P2}}=A_{1}B_{1}\left(\overline{A_{2}}+\overline{B_{2}}\right)$, (S42)

$Q_{\text{P3}}=\overline{A_{1}}A_{2}B_{1}+A_{1}\overline{B_{1}}B_{2}+A_{1}B_{1}\left(\overline{A_{2}}B_{2}+A_{2}\overline{B_{2}}\right)$, (S43)

$Q_{\text{P4}}=A_{2}B_{2}$. (S44)

It should be noted that the output *Q*_P3_ comprises an identical number of switches in Eq. (S39) and Eq. (S43). The latter is employed in the construction of the 2-bit multiplier, as it is more appropriate for our polygonal modules. The prototype and test of the 2-bit multiplier on an octagonal module with 22 switches in total are presented in Figure S13d and Movie S4. For instance, when the mechanical inputs *A* = (1 0), *B* = (1 1), the binary output of the 2-bit multiplier is (0 1 1 0), which conforms to the arithmetic logic of 2×3=6 in decimal.

Moreover, Figure 3f-h and Figure S14 present a 2-bit divider on an octagonal module, which performs the division between two operands *A*(*A*_1_ *A*_2_)_2_ and *B*(*B*_1_ *B*_2_)_2_. This yields the resulting binary quotient *Q*_Quot_(*Q*_Q1_ *Q*_Q2_)_2_ and remainder *Q*_Rema_(*Q*_R1_ *Q*_R2_)_2_, with the output *Error* representing a mathematical error when the divisor *B*(*B*_1_ *B*_2_)_2_ = (0 0). According to the logic diagram and truth table in Figure 3f and Figure S14a, the SSoP functions of the 2-bit divider for each output can be derived as:

$Q_{\text{Q1}}=A_{1}\overline{A_{2}}\overline{B_{1}}B_{2}+A_{1}A_{2}\overline{B_{1}}B_{2}$, (S45)

$Q_{\text{Q2}}=\overline{A_{1}}A_{2}\overline{B_{1}}B_{2}+A_{1}\overline{A_{2}}B_{1}\overline{B_{2}}+A_{1}A_{2}\overline{B_{1}}B_{2}+A_{1}A_{2}B_{1}\overline{B_{2}}+A_{1}A_{2}B_{1}B_{2}$, (S46)

$Q_{\text{R1}}=A_{1}\overline{A_{2}}B_{1}B_{2}$. (S47)

$Q_{\text{R2}}=\overline{A_{1}}A_{2}B_{1}\overline{B_{2}}+\overline{A_{1}}A_{2}B_{1}B_{2}+A_{1}A_{2}B_{1}\overline{B_{2}}$, (S48)

$Error=\overline{B_{1}}\overline{B_{2}}$. (S49)

After being simplified by the QM algorithm, the corresponding QMSoP functions are as follows:

$Q_{\text{Q1}}=A_{1}\overline{B_{1}}B_{2}$, (S50)

, (S51)

$Q_{\text{R1}}=A_{1}\overline{A_{2}}B_{1}B_{2}$, (S52)

$Q_{\text{R2}}=\overline{A_{1}}A_{2}B_{1}+A_{2}B_{1}\overline{B_{2}}$, (S53)

$Error=\overline{B_{1}}\overline{B_{2}}$. (S54)

Next, the PCSoP functions for outputs *Q*_Q1_, *Q*_Q2_, *Q*_R1_, *Q*_R2_, and *Error* are obtained by a common factor extraction method, as follows

$Q_{\text{Q1}}=A_{1}\overline{B_{1}}B_{2}$, (S55)

, (S56)

$Q_{\text{R1}}=A_{1}B_{1}\overline{A_{2}}B_{2}$, (S57)

$Q_{\text{R2}}=\overline{A_{1}}B_{1}A_{2}+B_{1}A_{2}\overline{B_{2}}$, (S58)

$Error=\overline{B_{1}}\overline{B_{2}}$. (S59)

The designed 2-bit divider using PCSoP functions requires 22 switches in total, see Figure 3g and Figure S14b. Its prototype and experimental results are illustrated in Figure 3h, Figure S14c, and Movie S5.

**S6. The PCSoP-based automated implementation method functions for an arbitrary logic operator in MATLAB**

This section introduces the methodology for implementing an arbitrary logic operator with known input-output correspondences. For ease of understanding, a 2-bit adder is used as an example. First, the program automatically generates the combination of two input operands *A*(*A*_1_ *A*_2_) and *B*(*B*_1_ *B*_2_), and the corresponding binary output *Q*_Add_(*Q*_Cout_ *Q*_S1_ *Q*_S2_), as illustrated in Table S1. We then process each output *Q*_Cout_, *Q*_S1_, and *Q*_S2_ individually, using the relatively complex *Q*_S1_ as an example for illustration. Its SSoP function is derived from the truth table, representing the sum of the minterms for each output bit. Specifically, we select all inputs corresponding to *Q*_S1_=1, as shown in Table S2. Each minterm contains the product of all input Boolean terms, where if the input *A*_1_ is 0, the inverse of the input $\bar{A_{1}}$ is included in the product, and vice versa. Consequently, the SSoP function of output *Q*_S1_ is Eq. (S12).

Since each Boolean term in the function corresponds to a switch within the circuit, simplifying the SSoP function is equivalent to reducing the number of switches, thereby minimizing the scale of the mechanical metamaterial. The QM algorithm ^[44]^ is initially used for this simplification, with the simplified inputs as shown in Table S3, where '-' denotes input terms that have been consolidated and removed, i.e., switches that can be omitted. Thus, the QMSoP function of output *Q*_S1_ is changed to Eq. (S15).

Furthermore, the concept of temporal parallelism in parallel computing ^[41,42]^ can be applied to the QMSoP functions because the polygonal module allows the bifurcation of signal transmission. This implies the possibility of one input to multiple outputs, so we can reduce the QMSoP function to the PCSoP function through a common factor extraction method. The corresponding logic circuits of the extracted common factors can pass their outputs to multiple subsequent circuits. Since binary addition typically sums the bits of two operands at corresponding positions and then decides whether to perform a carry operation based on the result, here we place the bits of the two operands at adjacent positions to facilitate the extraction of common factors, see Table S4.

The common factors should only be extracted from the first column *A*_1_ of the table to the right columns in sequence or from the last column *B*_2_ to the left in sequence, otherwise, output errors will occur. Rows with suitable common factors should be compared and placed in adjacent positions, with two additional columns inserted to the right. Adjacent rows with common factors should be given the same row number, and the length of their common factor should be noted, with common factors as positive from left to right and vice versa. Each row is compared with every other row to record the length of the common factor (0, 1, 2, ..., -1, -2, ...), and the two rows with the largest absolute value of common factor length are selected as adjacent, with their row numbers and common factor lengths assigned. Iterating from the first row to the last row completes one round of iteration, resulting in Table S5. For instance, the second and third rows have identical values in columns *A*_1_ and *B*_1_, allowing a common factor to be extracted. Since the output *Q*_S1_ is the second bit in the binary output *Q*_Add_(*Q*_Cout_ *Q*_S1_ *Q*_S2_), its row numbers start from 2.1, i.e., these rows are assigned the identical row number 2.1. The length of the common factor *A*_1_*B*_1_ is 2, as it is extracted from left (*A*_1_) to right. At this point, the function of output *Q*_S1_ is reduced to

$Q_{\mathrm{S}1}=\overline{A_{1}}B_{1}\left(\overline{A_{2}}+\overline{B_{2}}\right)+A_{1}\overline{B_{1}}\left(\overline{A_{2}}+\overline{B_{2}}\right)+\left(\overline{A_{1}}\overline{B_{1}}+A_{1}B_{1}\right)A_{2}B_{2}$. (S60)

Next, contiguous rows with identical row numbers are treated as a row group, and the process of identifying common factors is repeated. In this case, common factors must be extracted in the opposite direction to the common factors extracted previously. For example, in Table S5, row groups 2.1 and 2.3 have identical input combinations for *A*_2_ and *B*_2_. Therefore, these row groups are placed next to each other, and the row number of group 2.3 is re-designated as 2.101, indicating that it has a common factor with row 2.1 that spans a length of -2, see Table S6. And the function of output *Q*_S1_ is reduced to

$Q_{\mathrm{S}1}=\left(\overline{A_{1}}B_{1}+A_{1}\overline{B_{1}}\right)\left(\overline{A_{2}}+\overline{B_{2}}\right)+\left(\overline{A_{1}}\overline{B_{1}}+A_{1}B_{1}\right)A_{2}B_{2}$. (S61)

In addition, row groups that share the same first two digits of their identifiers are aggregated into a larger composite group. Within these groups, common factors are re-examined in the reverse direction of the previous iteration. This iterative process is repeated sequentially until no more common factors can be extracted, culminating in the final input matrix, and thus the PCSoP function is derived. Using the row number information and common factor lengths from the table, our algorithm detects the presence of common factors and calculates the final number of switches used. The final input-output configuration for the 2-bit adder is delineated in Table S7, with a total requirement of 22 switches.

**S7. The comparison of the number of units used in mechanical logic, computing, information display and recognition between our work and existing ones**

The computational utility of a single metamaterial can be enhanced by allowing multiple mechanical logic networks to co-exist in the same structure^1^. However, regarding logic gates, different units or structures were typically used for different gates in prior works (Figure 5 and Table S8). Refs ^[5,6,8,10,11,14,16,21-26,28,30-35,39]^ construct various units that partially or fully implement logic gates such as NOT, OR, AND, NOR, NAND, XOR, and XNOR through various assembly and interconnection methods, as shown in Figure 5 with 30% transparent diamonds. For instance, ref. ^[22]^ constructs the OR, AND, NOR, NAND, XOR, and XNOR logic gates using two units each. Recent work has developed to implement multiple functions on a single structure, as in ref. ^[35]^, where three logic gates, NOT, OR, and AND, are implemented sequentially on the same mechanical transistor by thermal reconfiguration, as shown in Figure 5, where the corresponding rhombus at the bottom spans the intervals of NOT, OR, and AND gates. Similarly, Table S8 illustrates these situations by representing these logic gates in the same table cell. In addition, switching of NOR and NAND gates was achieved on a 3-unit structure by tuning the magnitude of the applied excitation ^[34]^; six dual-input logic gates were implemented sequentially on a 10-unit platform with different circuit arrangements ^[33]^. Our approach uses a quadrilateral module composed of four 4-bar-Sarrus linkage units to simultaneously achieve NOT, OR, AND, NOR, NAND, XOR, and XNOR gates, and this module can be further employed to implement a half adder/subtractor.

Furthermore, for complex mechanical computing systems that require numerous units, streamlining the system to reduce redundant units can further improve the computational utility and, consequently, the level of integration. The bottom-up design approach is suitable for constructing a universal design framework, e.g., ref. ^[23]^ achieves 2-bit arithmetic operators with up to 44 units and proposes a simplified universal design framework. However, due to the limited number of accessible interactions between gates, this framework has not yet been implemented in a structure with a minimalist design without redundant units, and it still contains some duplicate mechanical logic network components that need to be removed. In contrast, a top-down design approach, which emphasizes the importance of the structural design and layout, enables higher-level functionality like a 2-bit adder (only 10 units) ^[39]^ to be directly designed with fewer mechanism units, rather than assembling individual logic gates, thereby reducing system complexity. However, due to the lack of detailed design rules, top-down methods are better suited to logical operations by integer accumulation, such as addition and related information recognition (with 55 units), which naturally align with the motion of mechanical mechanisms. Subtraction and multiplication have not yet been developed in the top-down approach. Division, with its inherent discontinuity requiring division splitting rather than operand stacking, remains unexplored. Our polygonal module-based logical operators, with fewer units, match or exceed the functionality of existing designs, with an extendable design framework. We have implemented the full adder/subtractor on a hexagonal module and a 2-bit arithmetic operator that performs addition, subtraction, multiplication, or division on an octagonal module. We have also realized the information display for a BCD to 7-segment display decoder on a hexagonal module and synchronized recognition of three mathematical properties of the generated decimal numbers on an octagonal module.

**S8. Figures S1 to S19**


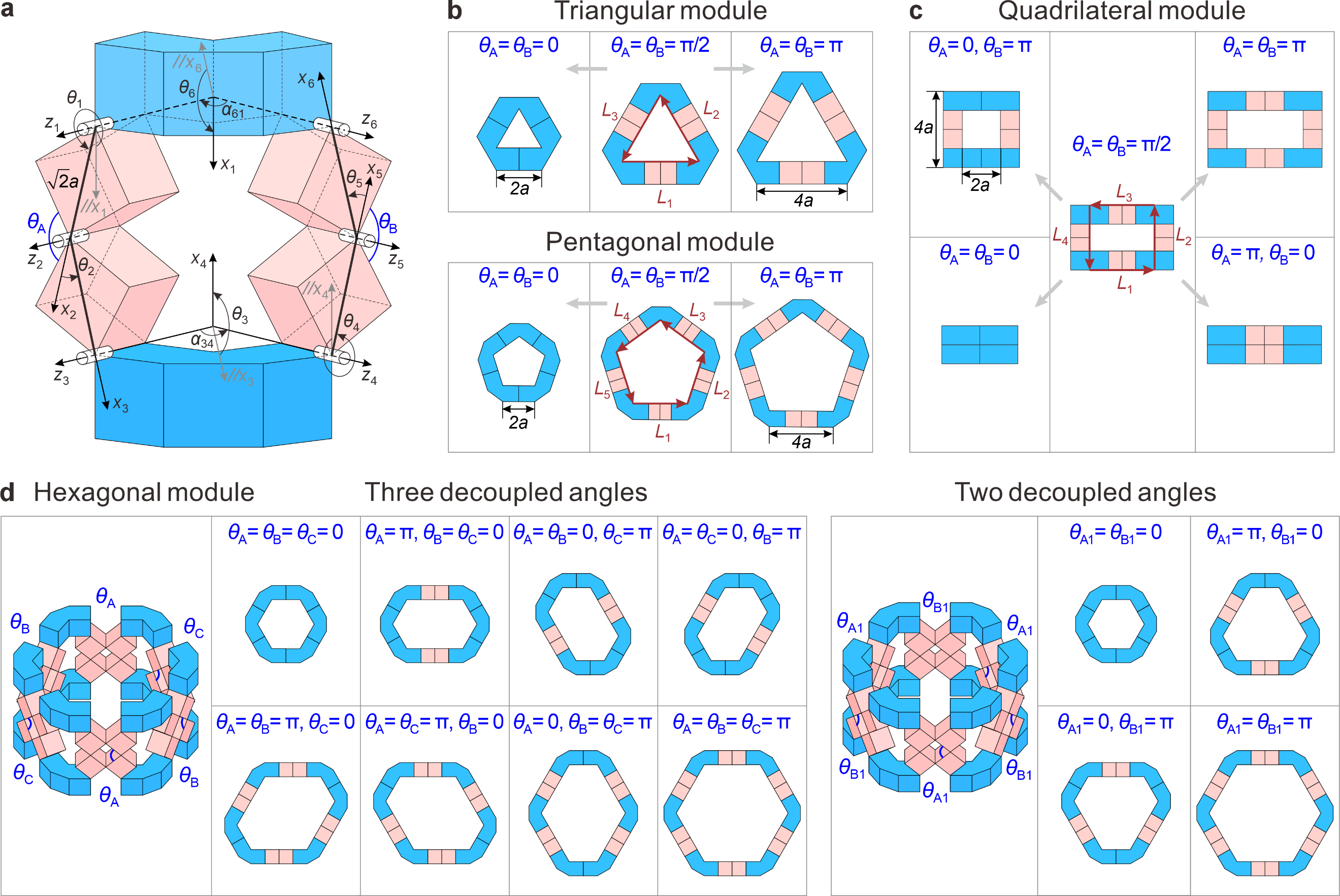


Figure S1. Kinematic analysis of the polygonal modules. a) Sarrus linkage with its schematic diagram in D-H notations. b) Triangular and pentagonal modules with only one motion path and no bifurcation. c) The quadrilateral module composed of four 4-bar Sarrus linkages with a kinematic bifurcation point and two motion paths. d) The bifurcation behaviors of the hexagonal module are divided into two groups on the basis of the number of decoupled dihedral angles. The first group has three decoupled dihedral angles *θ*_A_, *θ*_B_, and *θ*_C_ and 8 extreme configurations; the second group has two decoupled dihedral angles *θ*_A1_ and *θ*_B1_ and 4 extreme configurations.
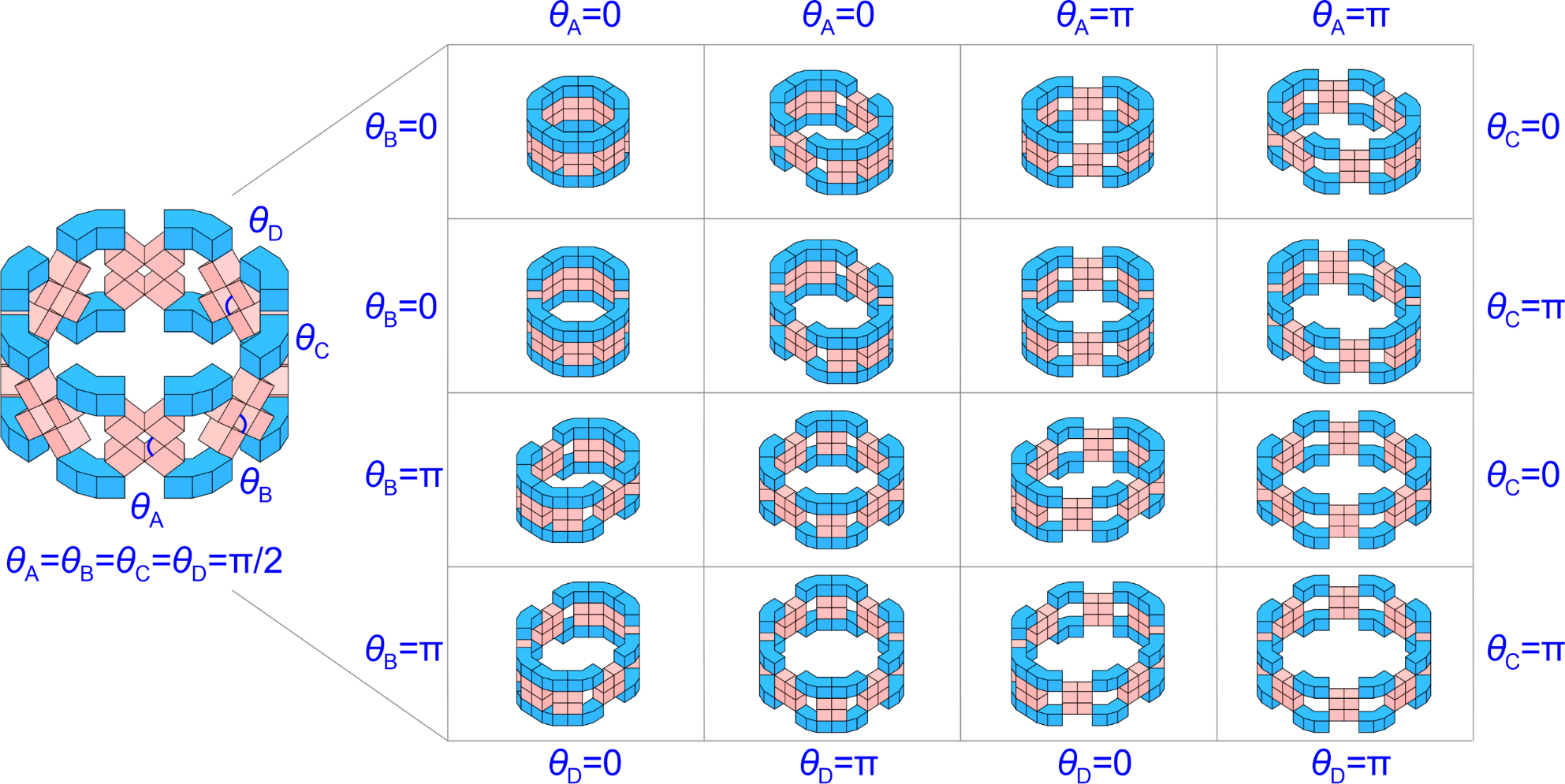


Figure S2. Octagonal module with four decoupled angles *θ*_A_, *θ*_B_, *θ*_C_, and *θ*_D_ and 16 extreme configurations.


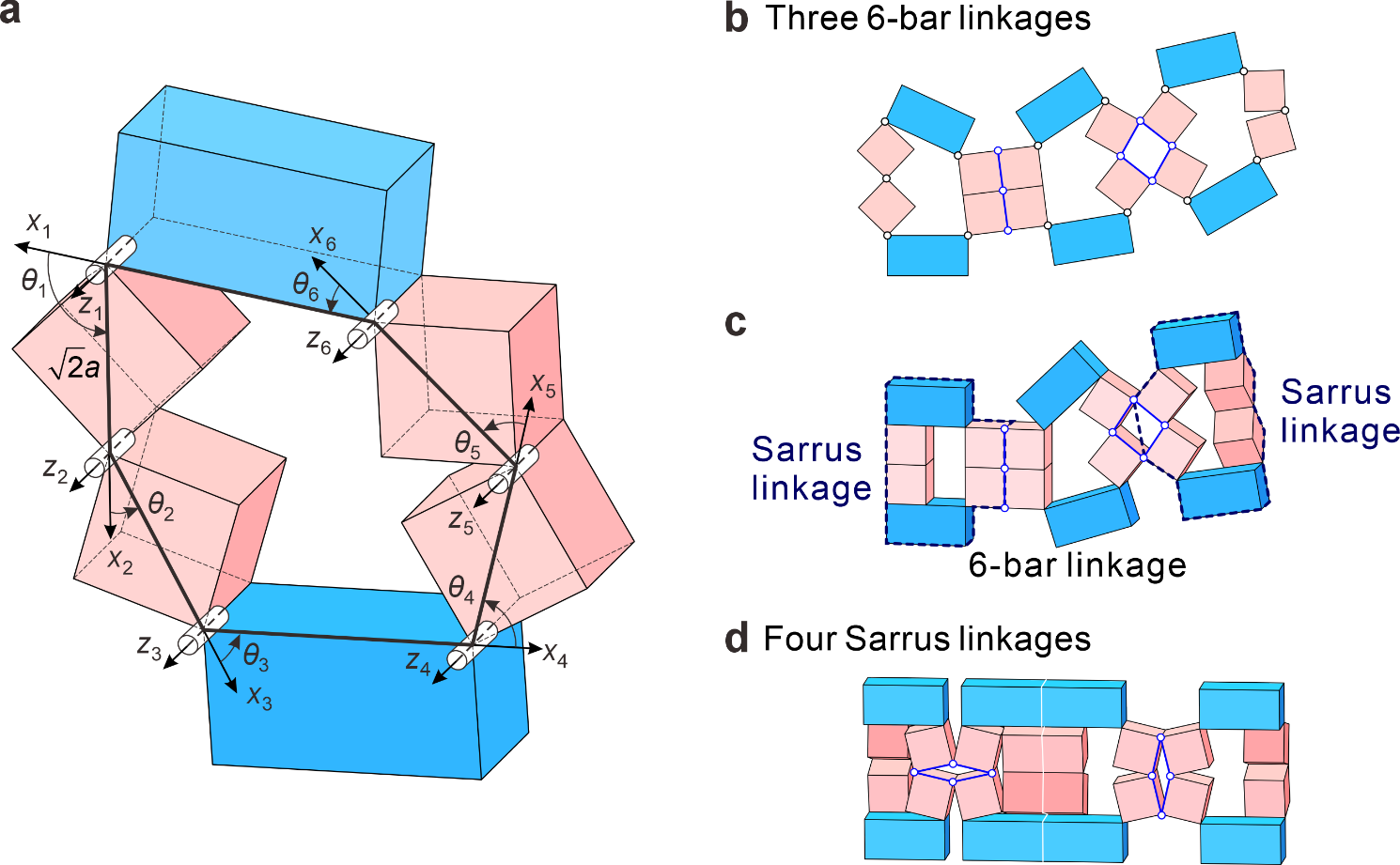


Figure S3. Metamaterials arranged in a straight line. a) A planar 6-bar linkage. b) A planar straight-line metamaterial consisting of three planar 6-bar linkages. c) A straight-line metamaterial consisting of two Sarrus linkages with a planar 6-bar linkage between them. d) A straight-line metamaterial composed of four Sarrus linkages, with the middle two merged by sharing side surfaces.


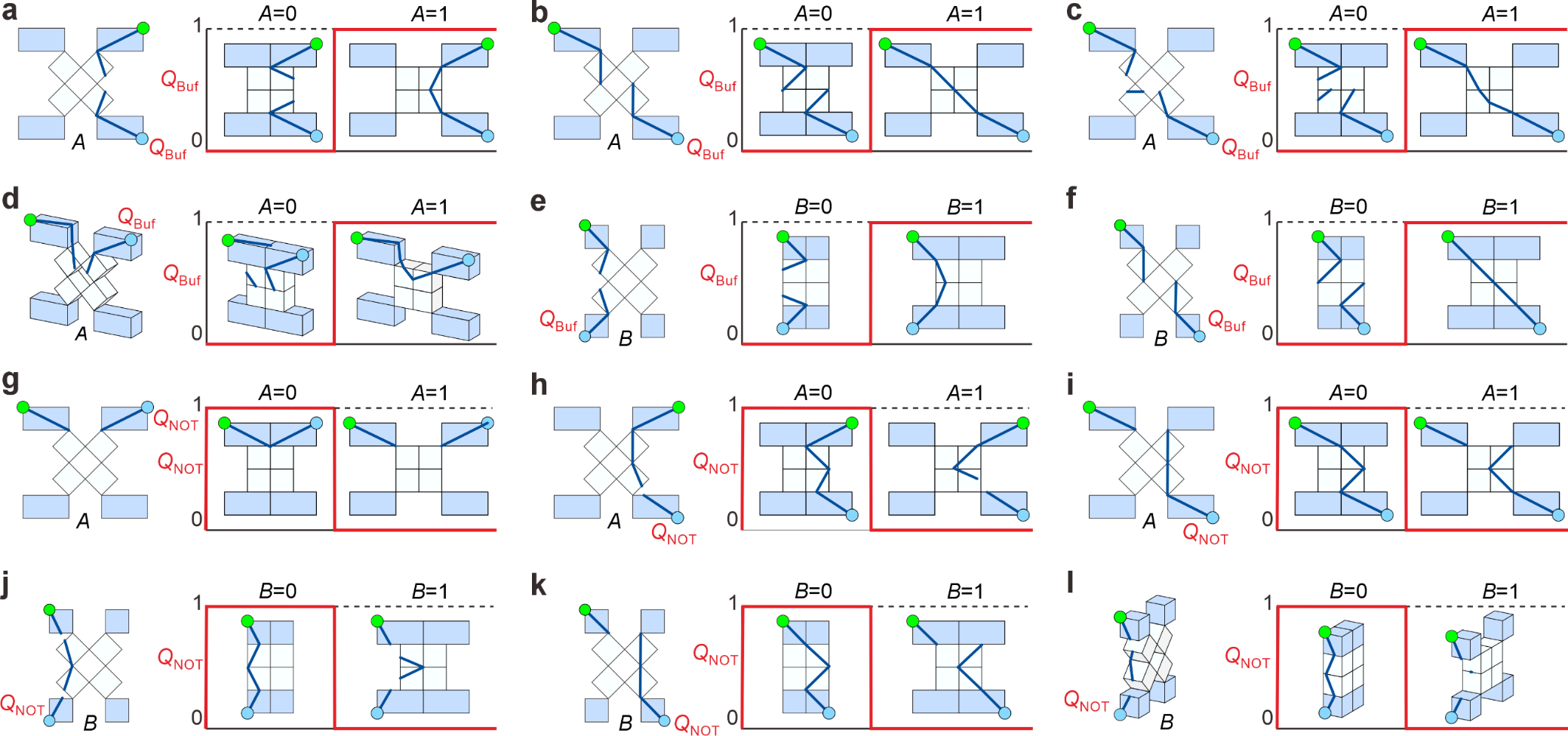


Figure S4. The design schematic of buffer and NOT logic gates in this paper. a-d) The buffer logic gates with input A. e, f) The buffer logic gates with input B. g-i) The NOT logic gates with input A. j-l) The NOT logic gates with input B.


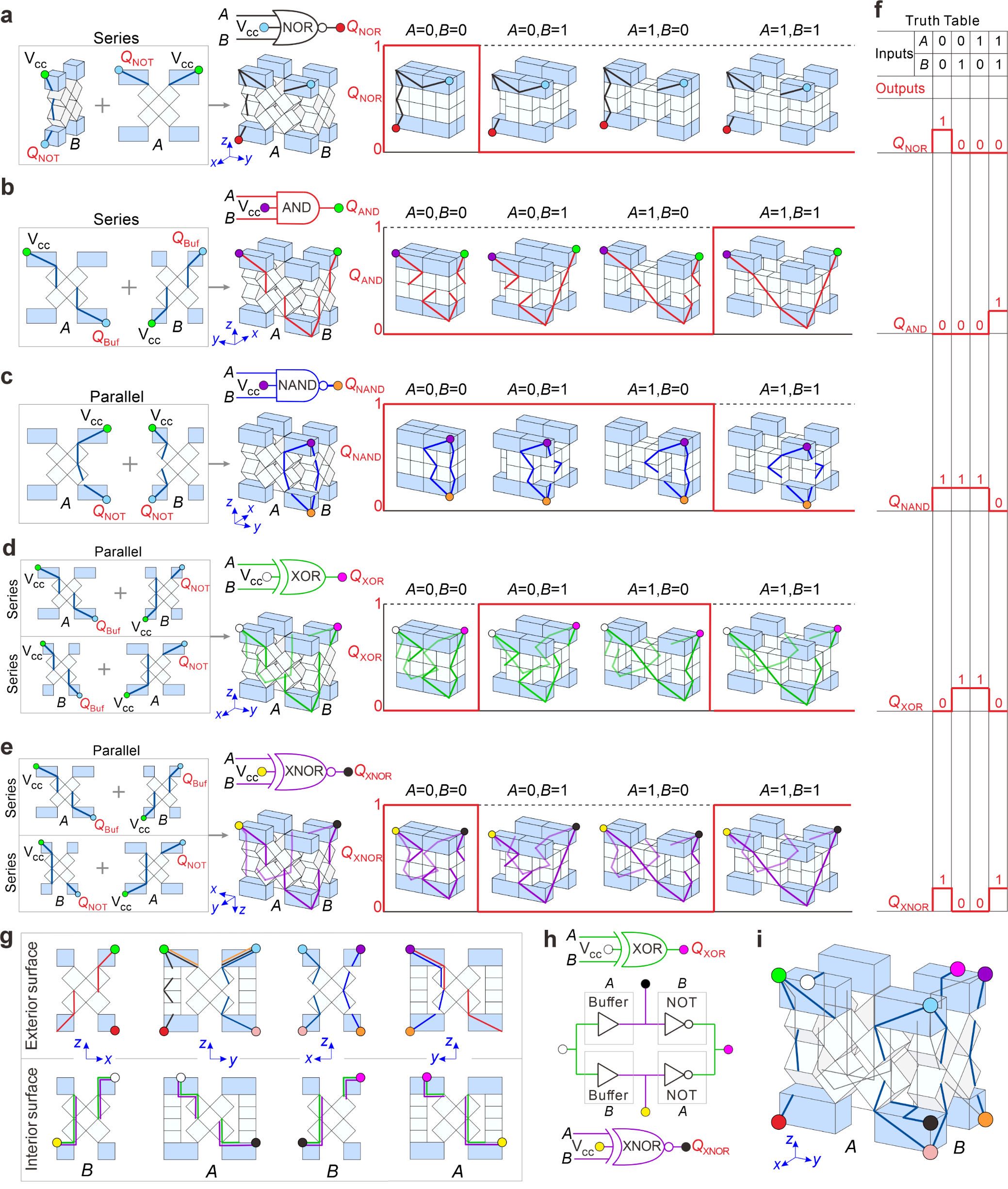


Figure S5. Design schematics of logic gates implemented on the quadrilateral module. a-e) Design schematics of the NOR, AND, NAND, XOR, and XNOR logic gates implemented on the quadrilateral module and their schematics in all cases. f) The truth table of the logic gates in (a-e). The circuits that are not visible from the current perspective are indicated in dark green in (d) and magenta in (e). g) Detailed diagrams of the exterior and interior surfaces of the integrated module. The circuits of the NOT, OR, NOR, AND, NAND, XOR and XNOR logic gates are denoted in orange, navy, black, red, blue, green and magenta respectively. h) Schematic diagram of the XOR and XNOR logic gates sharing a set of switch combinations. i) Integrated logic module with all seven logic gates.


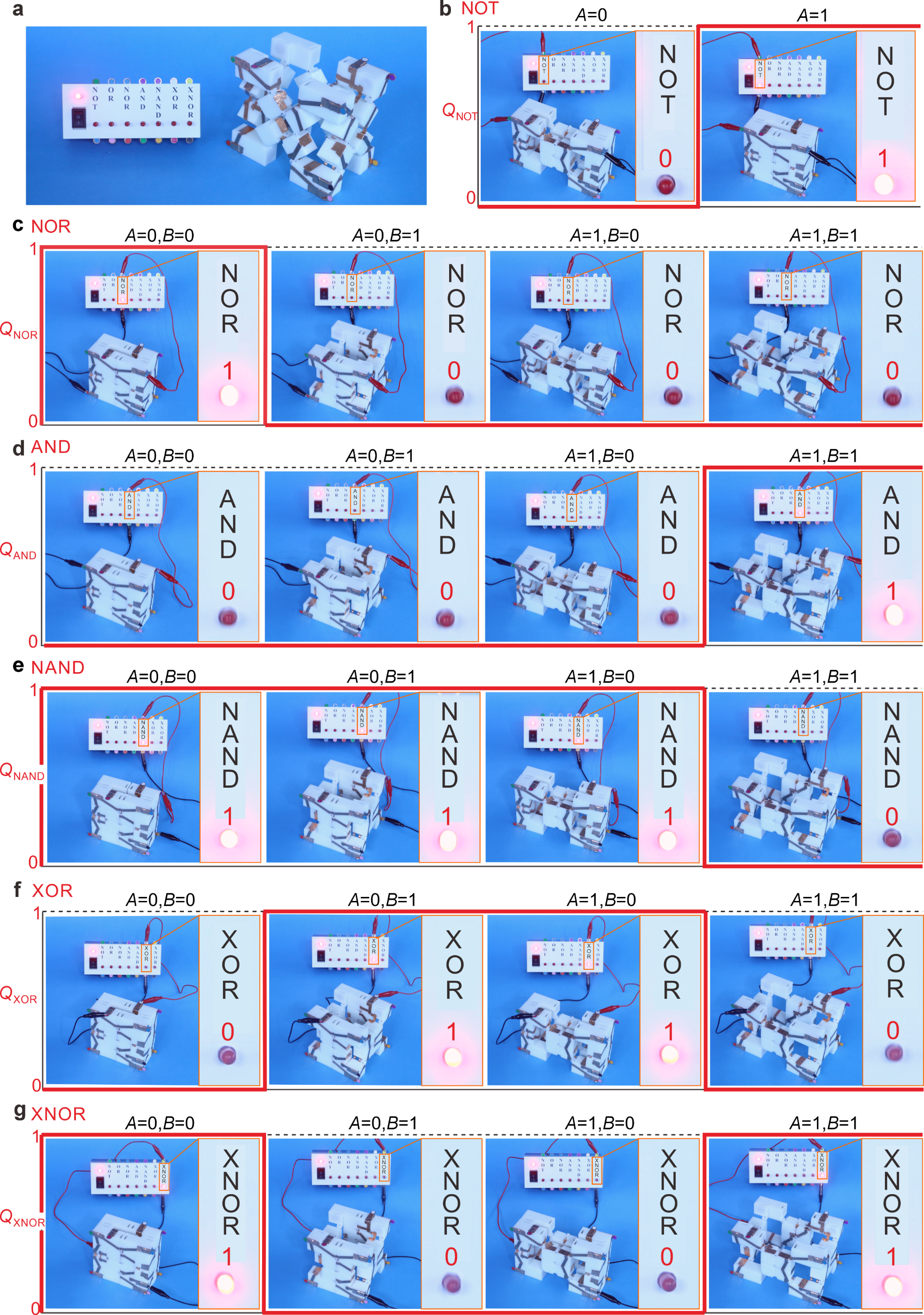


Figure S6. Experimental results of the integrated logic module in all the cases. a) Prototype of the integrated module with seven basic logic gates: NOT, OR, NOR, AND, NAND, XOR, and XNOR. b-g) Experimental results (red text) of the NOT, NOR, AND, NAND, XOR, and XNOR logic gates with different mechanical inputs (black text).


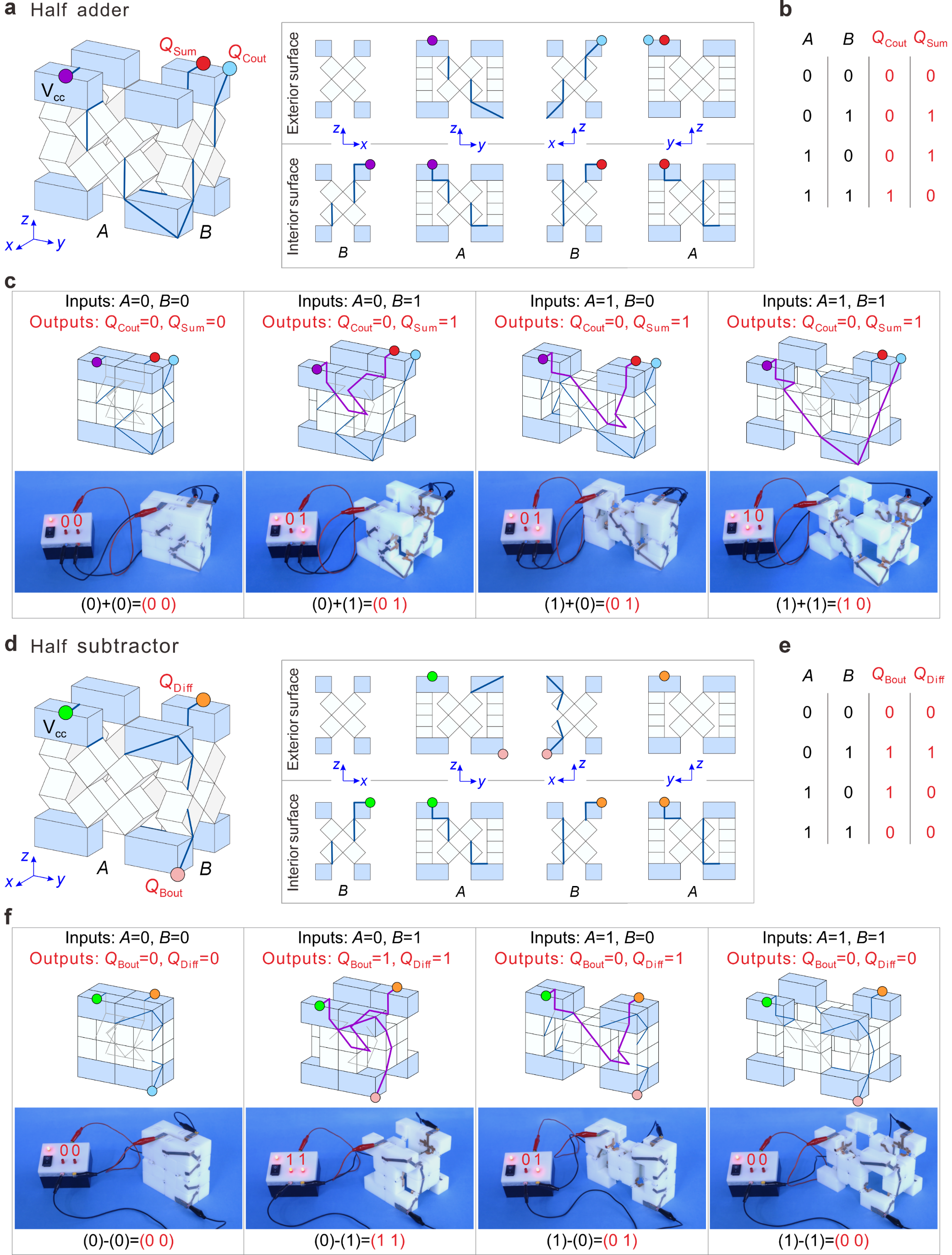


Figure S7. The half adder and half subtractor implemented on the quadrilateral module. a, b) Design of the half adder, with its truth table in (b). c) Schematics and experimental results of the half adder in all the cases. d, e) Design of the half subtractor, with its truth table in (e). f) Schematics and experimental results of the half subtractor in all the cases. Connected paths that result in an output of 1 are highlighted in purple, whereas lines that are not connected and are obscured from the current view are represented as thin grey lines.


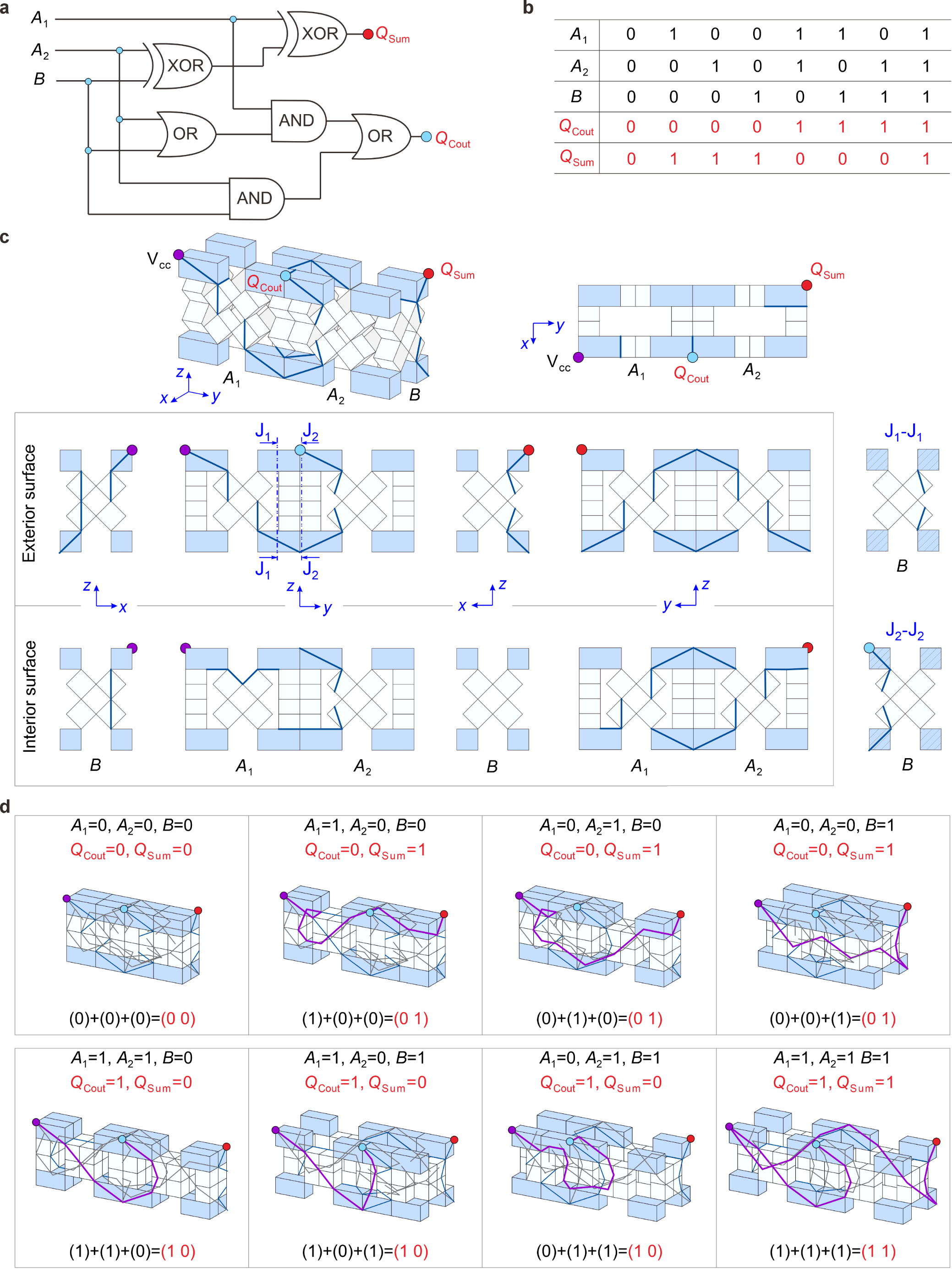


Figure S8. The full adder on the two modules in series. a, b) Logic diagram of the full adder with its corresponding (b) truth table. c) The design of the full adder. d) The simulation of the full adder in all cases. Connected paths that result in an output of 1 are highlighted in purple, while lines not connected and obscured from the current view are represented by grey thin lines.


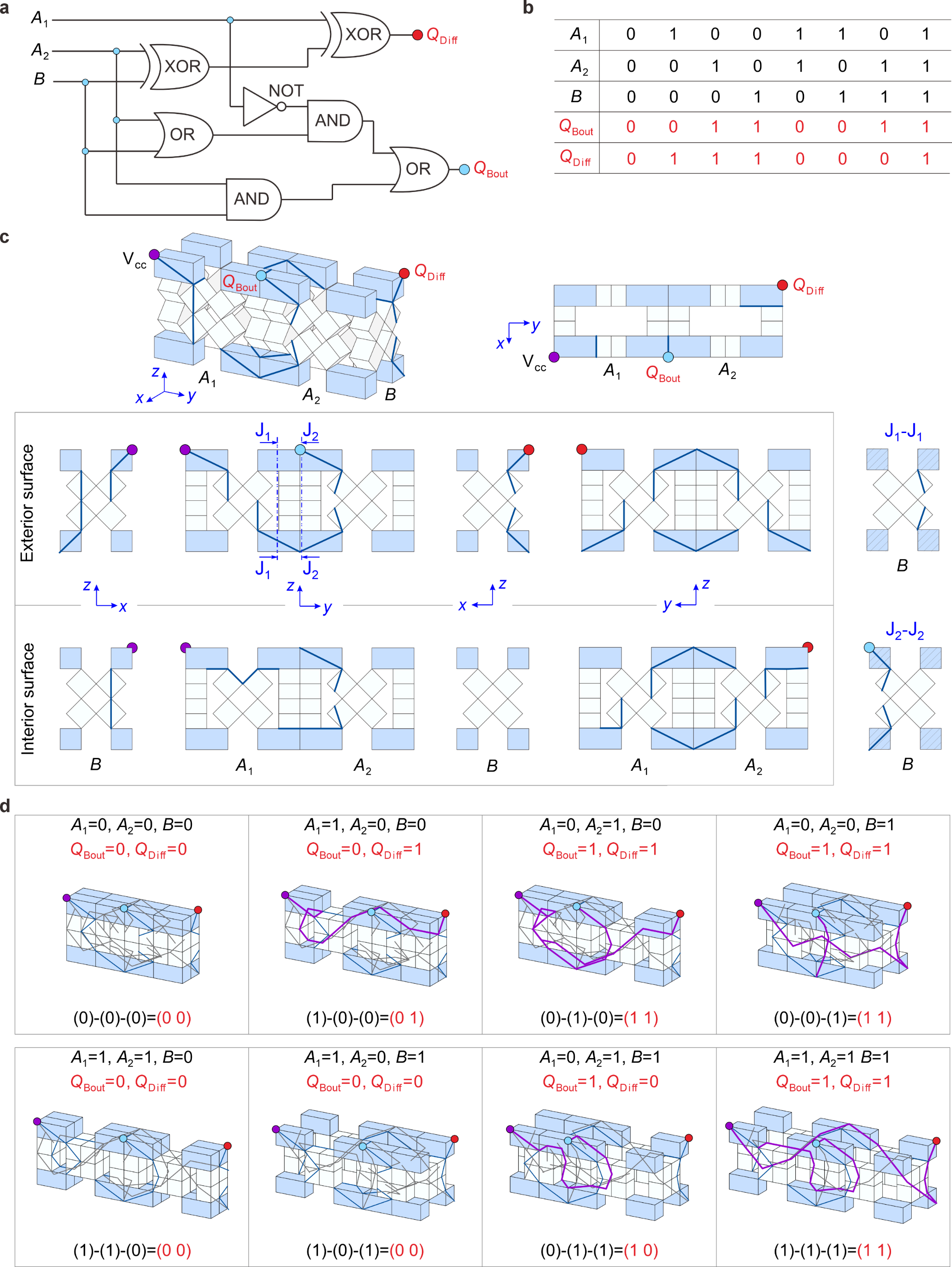


Figure S9. The full subtractor on the two modules in series. a, b) Logic diagram of the full subtractor with its corresponding (b) truth table. c) The design of the full subtractor. d) The simulation of the full subtractor in all cases. Connected paths that result in an output of 1 are highlighted in purple, while lines not connected and obscured from the current view are represented by grey thin lines.

**
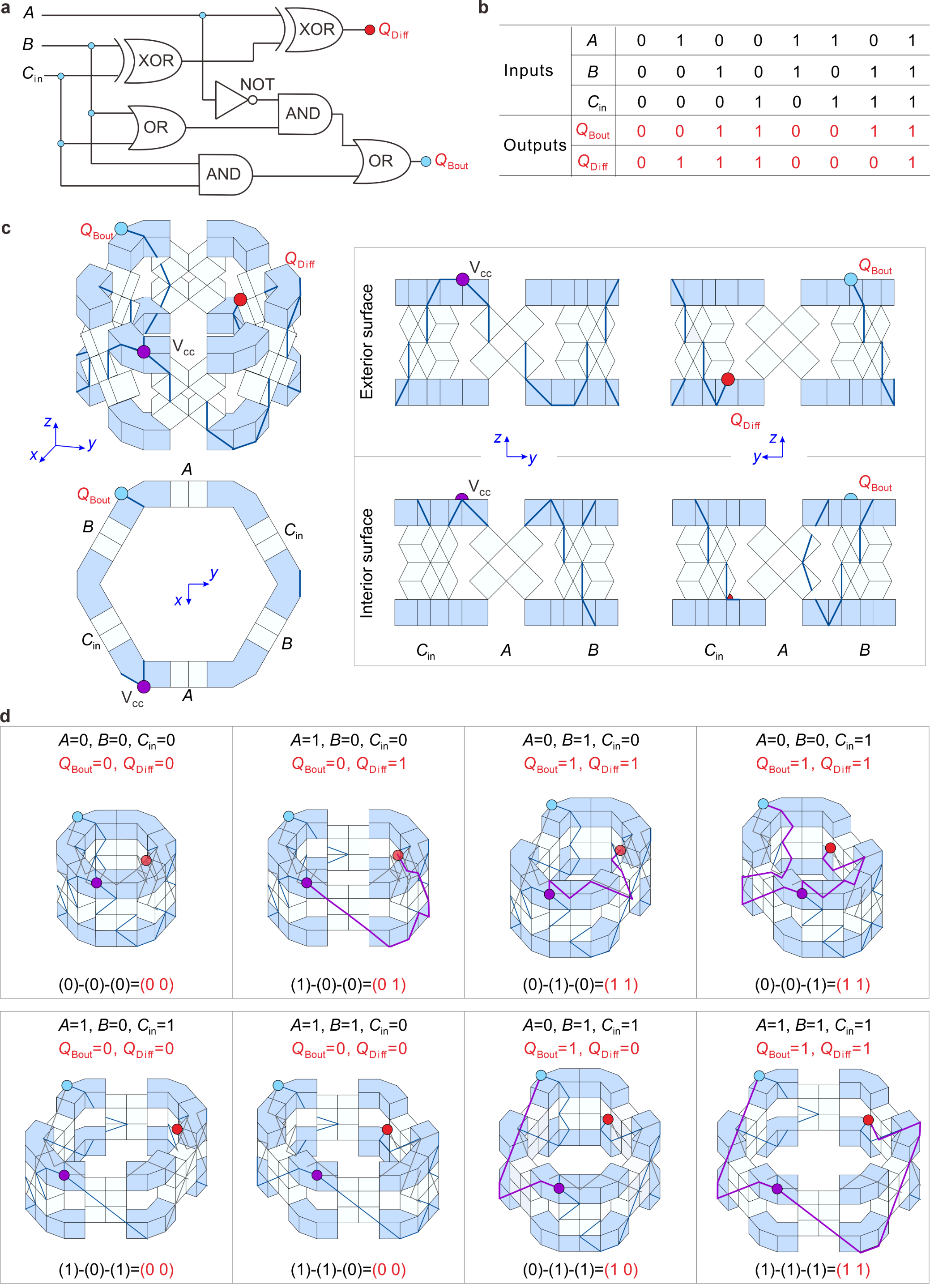
**

Figure S10. The full subtractor on a hexagonal module. a, b) Logic diagram of the full subtractor with its corresponding (b) truth table. c) The design of the full subtractor on a hexagonal module. d, The simulation of the full subtractor in all cases. Connected paths that result in an output of 1 are highlighted in purple, while lines not connected and obscured from the current view are represented by grey thin lines.


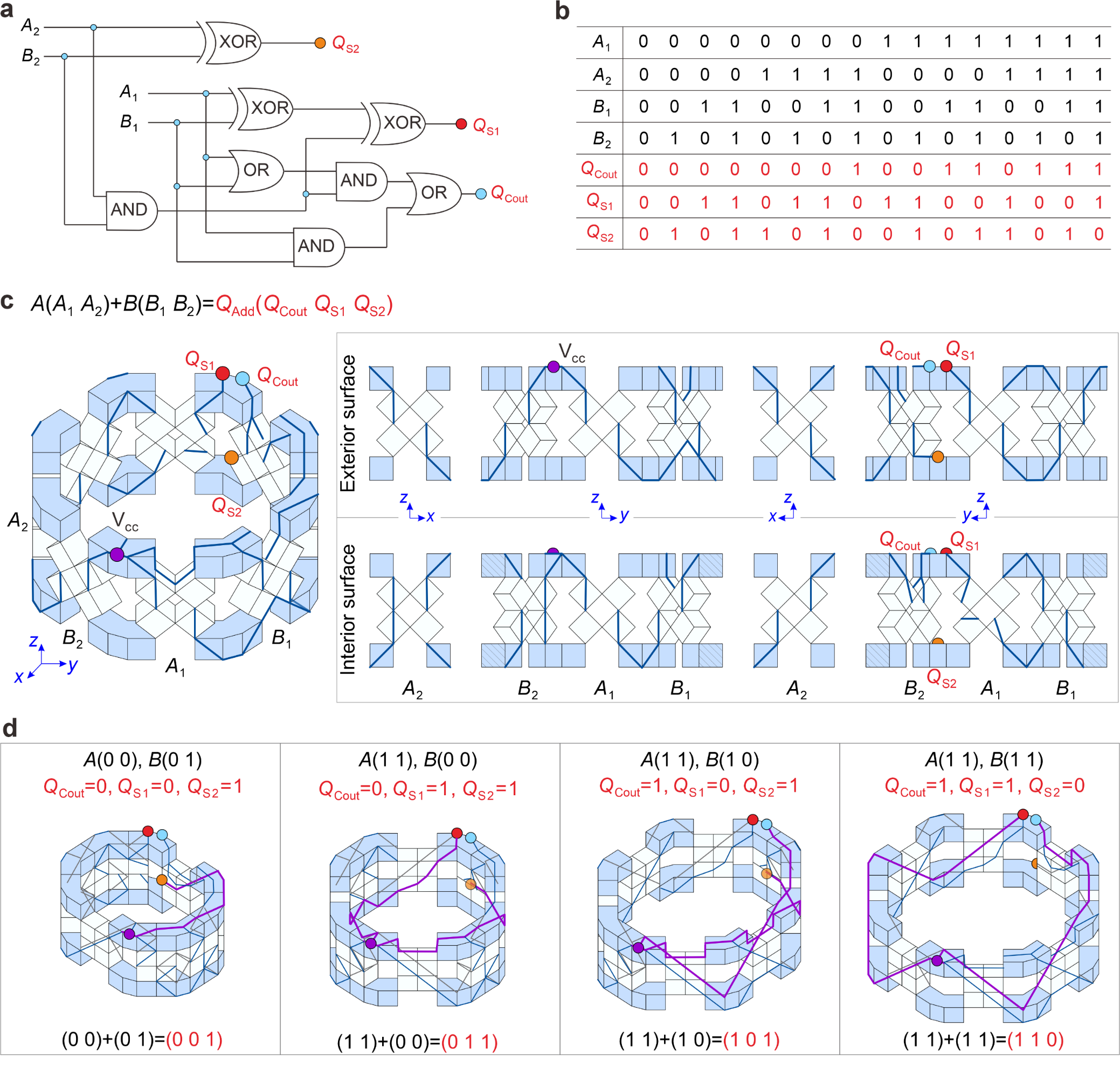


Figure S11. The 2-bit adder implemented on an octagonal module. a, b) Logic diagram of the 2-bit adder with its corresponding (b) truth table. c) Design of the 2-bit adder on the octagonal module. d) Schematics of the 2-bit adder in four cases. Connected paths that result in an output of 1 are highlighted in purple, whereas lines that are not connected and are obscured from the current view are represented by thin grey lines.


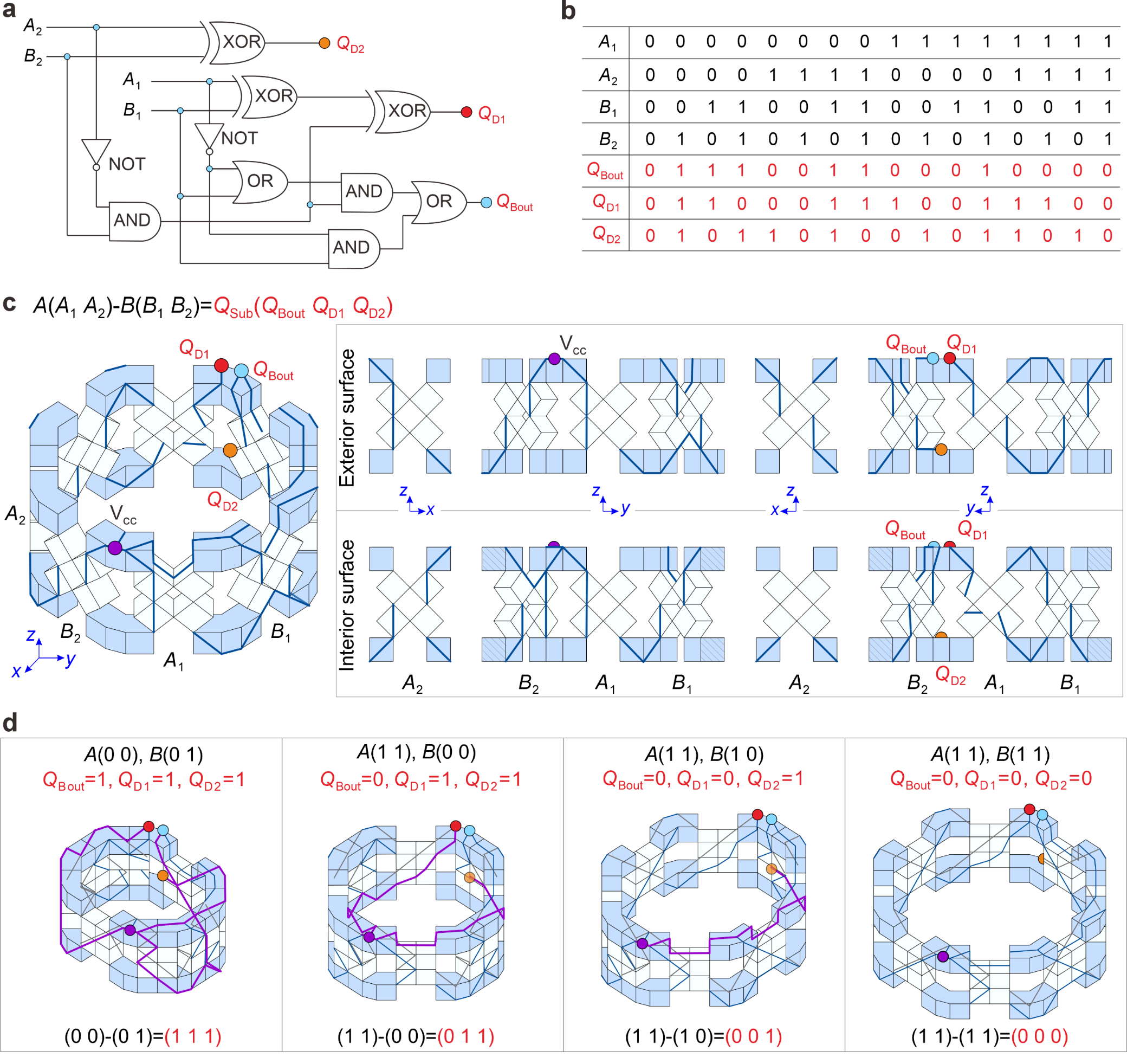


Figure S12. The 2-bit subtractor implemented on an octagonal module. a, b) Logic diagram of the 2-bit subtractor with its corresponding (b) truth table. c) Design of the 2-bit subtractor on the octagonal module. d) Schematics of the 2-bit subtractor in four cases. Connected paths that result in an output of 1 are highlighted in purple, whereas lines that are not connected and are obscured from the current view are represented by thin grey lines.


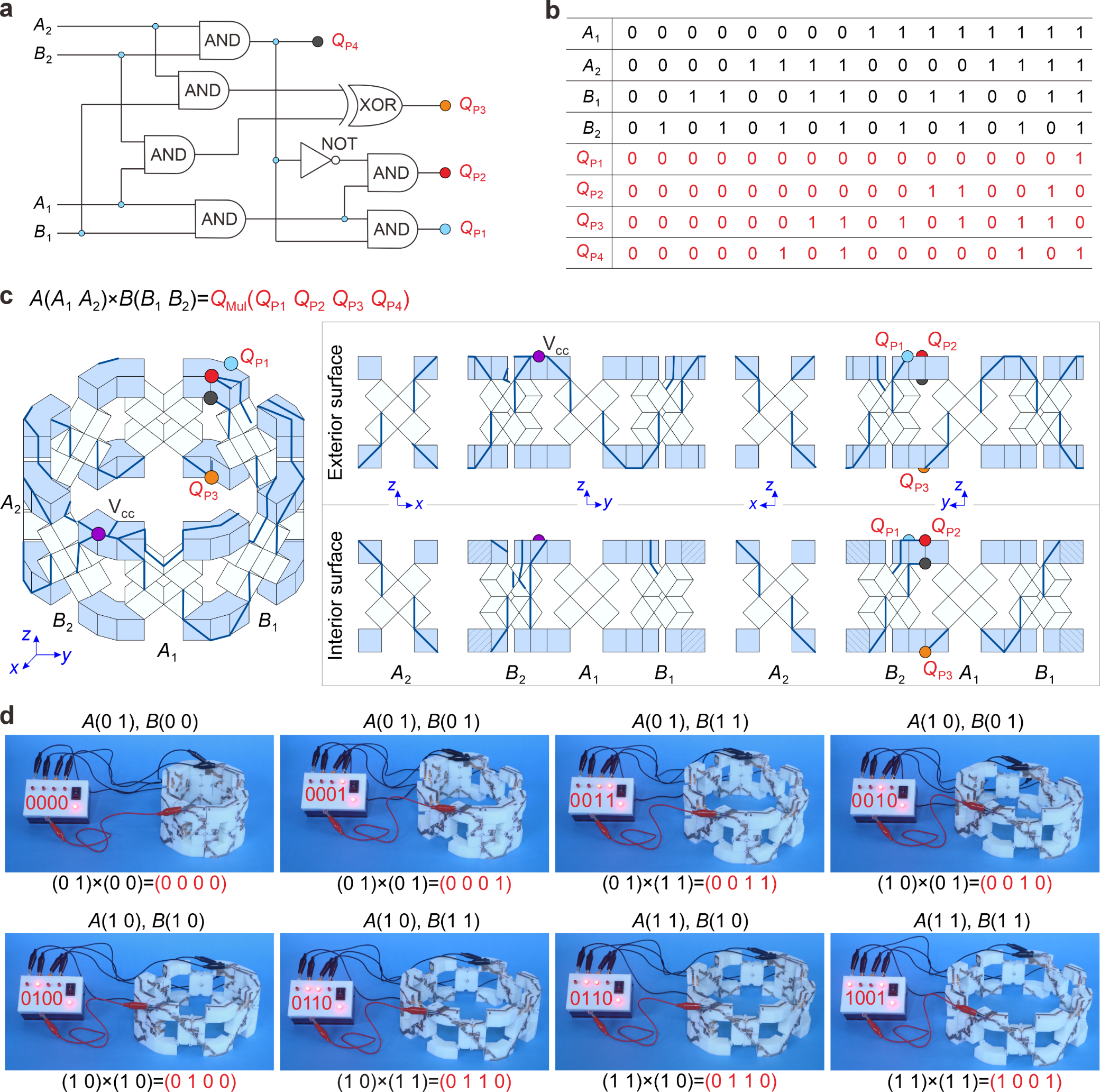


Figure S13. The 2-bit multiplier implemented on an octagonal module. a, b) Logic diagram of the 2-bit multiplier with its corresponding (b) truth table. c) Design of the 2-bit multiplier on an octagonal module. d) Experimental results of the 2-bit multiplier in eight cases.


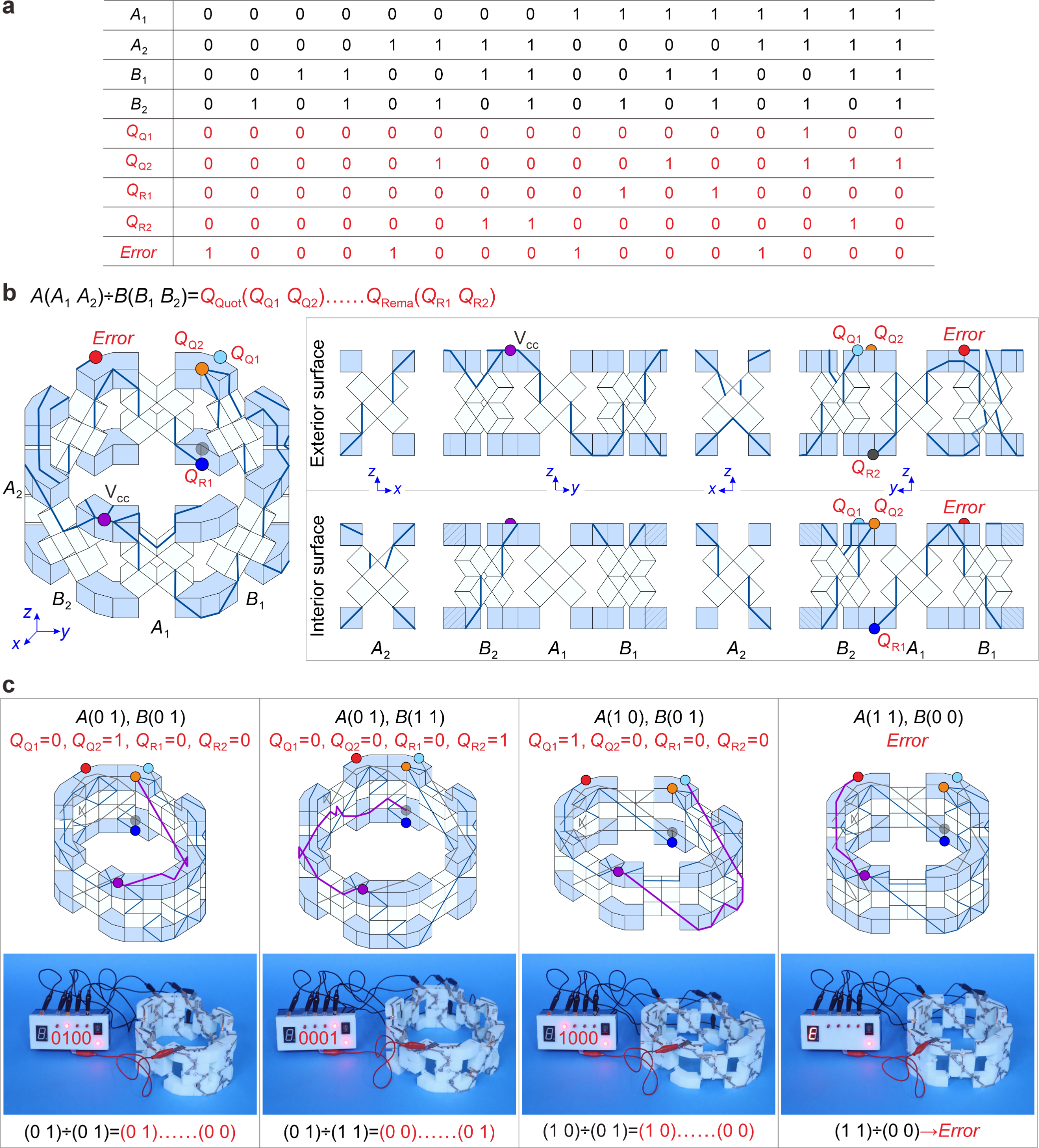


Figure S14. The 2-bit divider implemented on an octagonal module. a) The truth of the 2-bit divider. b) Design of the 2-bit divider on the octagonal module. c) Schematics and experimental results of the 2-bit divider in four cases. Connected paths that result in an output of 1 are highlighted in purple, whereas lines that are not connected and are obscured from the current view are represented by thin grey lines. If the divisor *B*(*B*_1_ *B*_2_)_2_ = (0, 0), the error-handling protocol triggers the output *Error*, which is displayed as the character '*E*' on a 7-segment display.


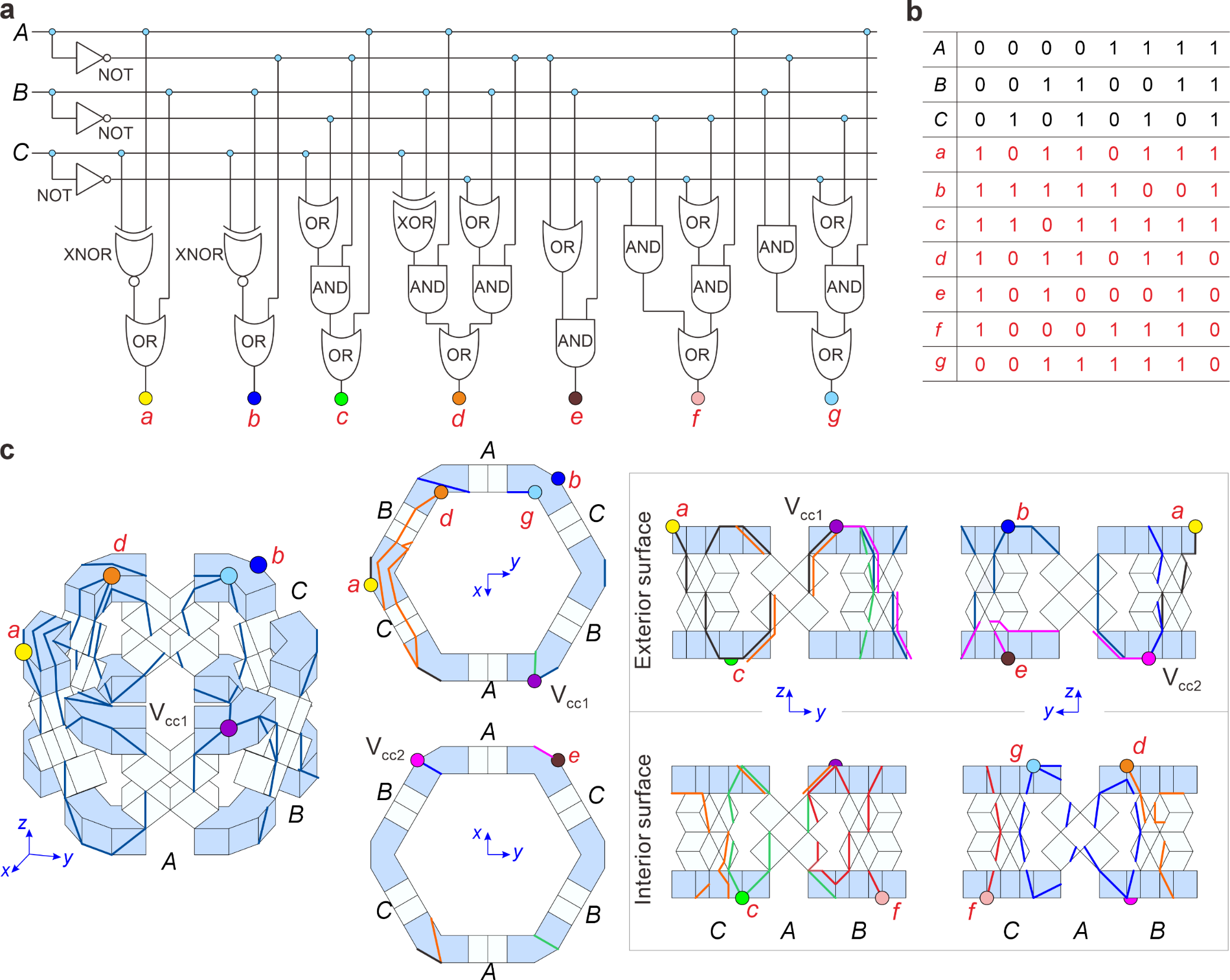


Figure S15. Hexagonal module-based binary-coded decimal (BCD) to 7-segment display decoder. a) Logic diagram of the BCD to 7-segment display decoder, with three mechanical inputs, *A*, *B*, and *C*, and seven outputs, *a*-*g*, represented by the yellow, blue, green, orange, brown, pink, and light blue nodes, respectively, with each output controlling a segment of the LED display. b) Illustration of the BCD to 7-segment display decoder. c) Design diagram of the BCD to 7-segment display decoder, with two power input terminals V_cc1_ and V_cc2_, denoted by the purple and magenta nodes, positioned at opposite ends of the hexagonal module to ensure a sufficient power supply. In the detailed view, the circuits corresponding to outputs a-g are represented by black, navy, green, orange, purple, red, and blue lines, respectively, whereas the circuits on the final module are denoted by navy lines.


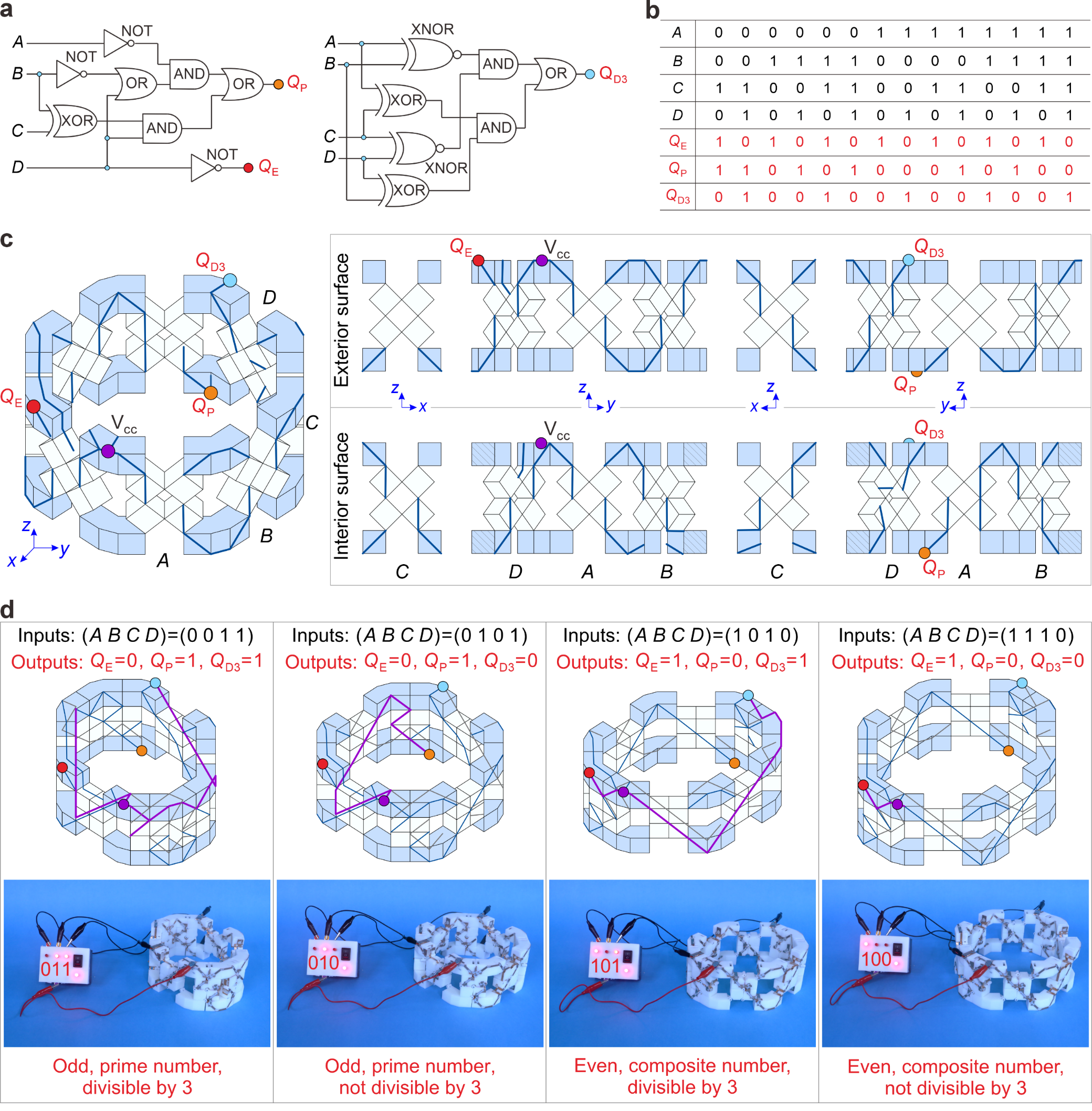


Figure S16. Recognition module for decimal numbers 2-15. a) Logic diagram of the recognition module. b) The truth table. c) Design diagram of the recognition module. d) Schematics and experimental results of the recognition module in four cases different from those in Figure 4f. Connected paths that result in an output of 1 are highlighted in purple, whereas lines that are not connected and are obscured from the current view are represented by thin grey lines.


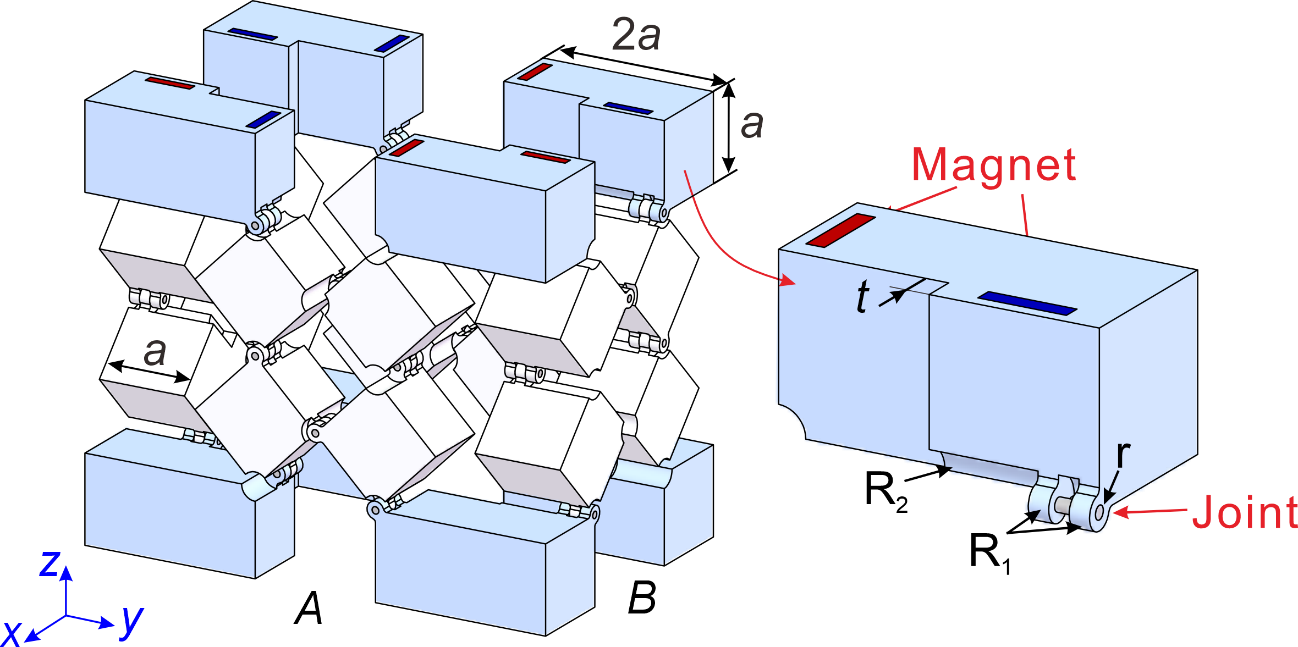


Figure S17. The schematic of the quadrilateral module with revolute joints.


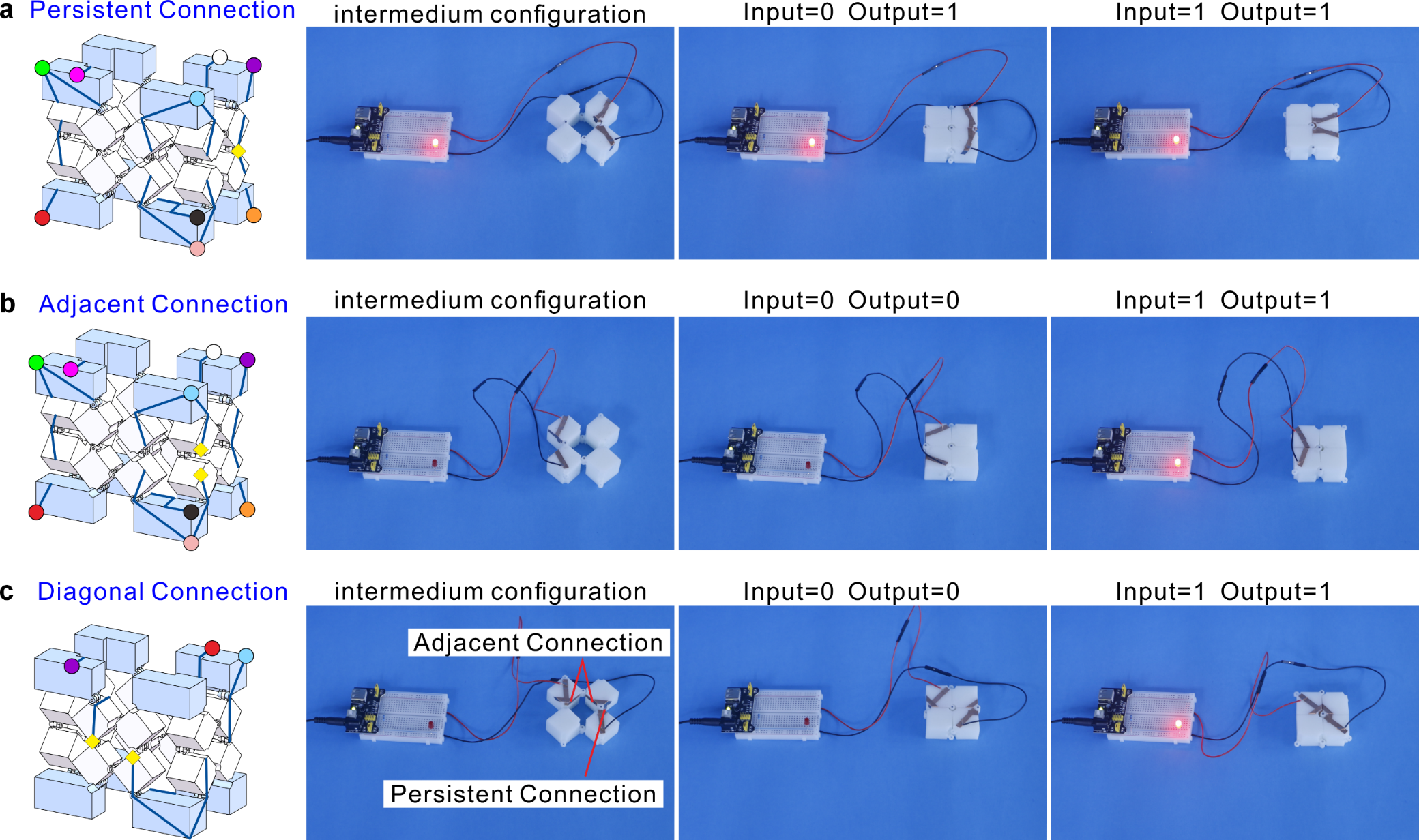


Figure S18. The three cases of circuit connection modes. a) The persistent connection. b) The adjacent connection. c) The diagonal connection. The related positions of these connections are represented by yellow diamonds.

**
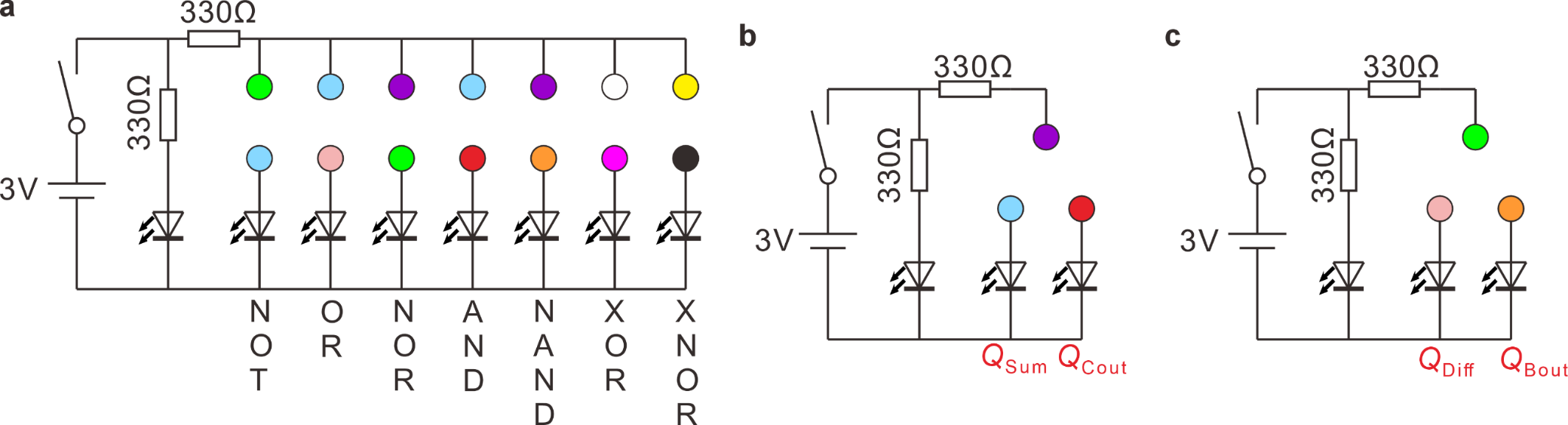
**

Figure S19. The circuit diagrams of the test boxes. a) The circuit diagram of the independent module integrated with all logic gates. b) The circuit diagram of the half adder. c) The circuit diagram of the half subtractor.

**S9. Tables S1 to S8**

Table S1. The input-output truth table of the 2-bit adder.

| *A*_1_ | *A*_2_ | *B*_1_ | *B*_2_ | *Q*_Cout_ | *Q*_S1_ | *Q*_S2_ |
| --- | --- | --- | --- | --- | --- | --- |
| 0 | 0 | 0 | 0 | 0 | 0 | 0 |
| 0 | 0 | 0 | 1 | 0 | 0 | 1 |
| 0 | 0 | 1 | 0 | 0 | 1 | 0 |
| 0 | 0 | 1 | 1 | 0 | 1 | 1 |
| 0 | 1 | 0 | 0 | 0 | 0 | 1 |
| 0 | 1 | 0 | 1 | 0 | 1 | 0 |
| 0 | 1 | 1 | 0 | 0 | 1 | 1 |
| 0 | 1 | 1 | 1 | 1 | 0 | 0 |
| 1 | 0 | 0 | 0 | 0 | 1 | 0 |
| 1 | 0 | 0 | 1 | 0 | 1 | 1 |
| 1 | 0 | 1 | 0 | 1 | 0 | 0 |
| 1 | 0 | 1 | 1 | 1 | 0 | 1 |
| 1 | 1 | 0 | 0 | 0 | 1 | 1 |
| 1 | 1 | 0 | 1 | 1 | 0 | 0 |
| 1 | 1 | 1 | 0 | 1 | 0 | 1 |
| 1 | 1 | 1 | 1 | 1 | 1 | 0 |

Table S2. The inputs corresponding to output *Q*_S1_=1.

| *A*_1_ | *A*_2_ | *B*_1_ | *B*_2_ |
| --- | --- | --- | --- |
| 0 | 0 | 1 | 0 |
| 0 | 0 | 1 | 1 |
| 0 | 1 | 0 | 1 |
| 0 | 1 | 1 | 0 |
| 1 | 0 | 0 | 0 |
| 1 | 0 | 0 | 1 |
| 1 | 1 | 0 | 0 |
| 1 | 1 | 1 | 1 |

Table S3. The inputs simplified by QM algorithm ^[44]^ corresponding to output *Q*_S1_=1.

| *A*_1_ | *A*_2_ | *B*_1_ | *B*_2_ |
| --- | --- | --- | --- |
| 0 | - | 1 | 0 |
| 0 | 0 | 1 | - |
| 0 | 1 | 0 | 1 |
| 1 | - | 0 | 0 |
| 1 | 0 | 0 | - |
| 1 | 1 | 1 | 1 |

Table S4. The inputs after exchanging columns.

| *A*_1_ | *B*_1_ | *A*_2_ | *B*_2_ |
| --- | --- | --- | --- |
| 0 | 1 | - | 0 |
| 0 | 1 | 0 | - |
| 0 | 0 | 1 | 1 |
| 1 | 0 | - | 0 |
| 1 | 0 | 0 | - |
| 1 | 1 | 1 | 1 |

Table S5. The inputs after one iteration.

| *A*_1_ | *B*_1_ | *A*_2_ | *B*_2_ | Row numbers | Length of common factors |
| --- | --- | --- | --- | --- | --- |
| 0 | 1 | - | 0 | 2.1 | 2 |
| 0 | 1 | 0 | - | 2.1 | 2 |
| 0 | 0 | 1 | 1 | 2.2 | -2 |
| 1 | 1 | 1 | 1 | 2.2 | -2 |
| 1 | 0 | - | 0 | 2.3 | 2 |
| 1 | 0 | 0 | - | 2.3 | 2 |

Table S6. The inputs after two iterations.

| *A*_1_ | *B*_1_ | *A*_2_ | *B*_2_ | Row numbers | Length of common factors |
| --- | --- | --- | --- | --- | --- |
| 0 | 1 | - | 0 | 2.1 | 2 |
| 0 | 1 | 0 | - | 2.1 | 2 |
| 1 | 0 | - | 0 | 2.101 | 2 |
| 1 | 0 | 0 | - | 2.101 | 2 |
| 0 | 0 | 1 | 1 | 2.2 | -2 |
| 1 | 1 | 1 | 1 | 2.2 | -2 |

Table S7. The input-output of the 2-bit adder using PCSoP functions.

| Output | *A*_1_ | *B*_1_ | *A*_2_ | *B*_2_ | Row numbers | Length of common factors |
| --- | --- | --- | --- | --- | --- | --- |
| *Q*_Cout_ | - | 1 | 1 | 1 | 1.1 | -2 |
| *Q*_Cout_ | 1 | - | 1 | 1 | 1.1 | -2 |
| *Q*_Cout_ | 1 | 1 | - | - | 1.2 | 0 |
| *Q*_S1_ | 0 | 1 | - | 0 | 2.1 | 2 |
| *Q*_S1_ | 0 | 1 | 0 | - | 2.1 | 2 |
| *Q*_S1_ | 1 | 0 | - | 0 | 2.101 | 2 |
| *Q*_S1_ | 1 | 0 | 0 | - | 2.101 | 2 |
| *Q*_S1_ | 0 | 0 | 1 | 1 | 2.2 | -2 |
| *Q*_S1_ | 1 | 1 | 1 | 1 | 2.2 | -2 |
| *Q*_S2_ | - | - | 0 | 1 | 3.1 | 2 |
| *Q*_S2_ | - | - | 1 | 0 | 3.1 | 2 |

Table S8. The comparison of the number of units used in mechanical logic, computing, information display and recognition between our work and existing ones.

|  | Mechanical logic | | | | | | | Computing | | | Information | |
| --- | --- | --- | --- | --- | --- | --- | --- | --- | --- | --- | --- | --- |
| Ref | NOT | OR | AND | NOR | NAND | XOR | XNOR | Half adder | Full adder | 2-bit operators | Display | Recognition |
| [5] | 3 | 10 | 6 | 6 | 10 | 10 | 10 | - | - | - | - | - |
| [6] | 1 | 2 | 2 | - | - | - | - | - | - | - | - | - |
| [8] | 4 | 4 | 2 | - | - | - | - | - | - | - | - | - |
| [14] | 1 | - | - | - | 2 | - | - | - | - | - | - | - |
| [16] | - | 1 | 1 | - | 3 | - | - | - | - | - | - | - |
| [21] | - | 2 | 2 | - | - | - | - | - | - | - | - | - |
| [22] | - | 2 | 2 | 2 | 2 | 2 | 2 | - | - | - | - | - |
| [25] | - | - | 3 | - | - | - | - | - | - | - | - | - |
| [26] | 4 | 4 | 4 | - | - | - | - | - | - | - | - | - |
| [28] | - | 9 | 9 | - | - | - | - | - | - | - | - | - |
| [30] | 1 | 1 | 1 | - | - | - | - | - | - | - | - | - |
| [31] | 1 | 1 | 1 | - | - | 1 | - | - | - | - | - | - |
| [24] | 2 | 4 | 3 | 3 | 4 | 2 | - | - | - | - | - | - |
| [33] | 3 | 10 | | | | | | - | - | - | - | - |
| [34] | 2 | 4 | 5 | 3 | | - | - | 32 | - | - | - | - |
| [35] | 1 | | | 2 | 2 | 2 | 2 | - | 7 | - | - | - |
| [10] | - | 4 | 4 | - | - | 8 | - | 12 | 36 | - | - | - |
| [11] | - | - | - | - | - | - | - | 6 | 15 | 26 | - | - |
| [23] | 1 | 10 | 10 | - | - | - | - | - | 21 | 44 | 64 | - |
| [32] | - | 4 | 4 | 4 | 4 | - | - | - | - | - | 25 | - |
| [39] | 2 | 1 | 2 | - | - | 2 | - | 3 | 5 | 10 | - | 55 |
| Our work | 4 | | | | | | | | 6 | 8 | 6 | 8 |

**S10. Legends for Movies S1 to S7**

Movie S1.

The quadrilateral logic module integrating seven basic logic gates.

Movie S2.

The half adder/subtractor on a quadrilateral module.

Movie S3.

The full adder on a hexagonal module.

Movie S4.

The 2-bit multiplier on an octagonal module.

Movie S5.

The 2-bit divider on an octagonal module.

Movie S6.

The binary-coded decimal to 7-segment display decoder on a hexagonal module.

Movie S7.

The recognition module for binary-coded decimal numbers 2-15 on an octagonal module.

**References**

[51] J. Denavit, R. S. Hartenberg, A kinematic notation for lower-pair mechanisms based on matrices, **1955**, 214-221.
